# Supplementary material for: Scalable carbon dioxide electroreduction coupled to carbonylation chemistry
Source: Nat Commun. 2017 Sep 8;8:489. doi: 10.1038/s41467-017-00559-8 (PMC5591205; doi:10.1038/s41467-017-00559-8)
Supplement: Supplementary file 1 — Supplementary Information [file 41467_2017_559_MOESM1_ESM.pdf]

a

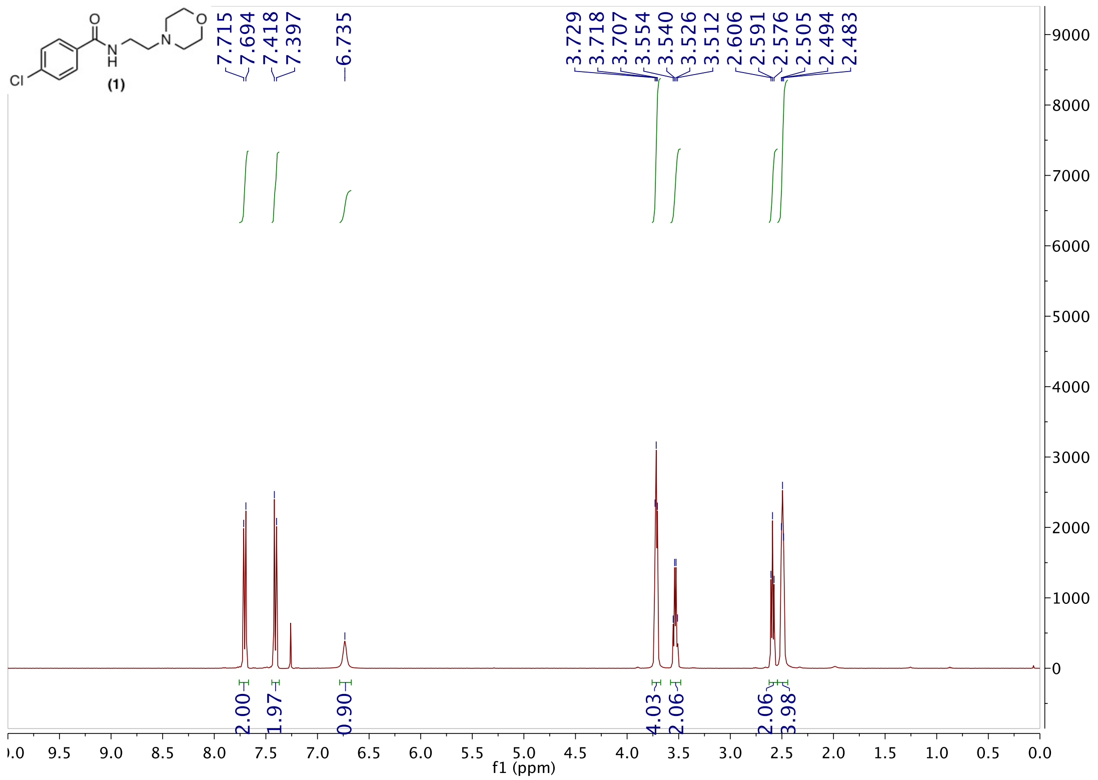

b

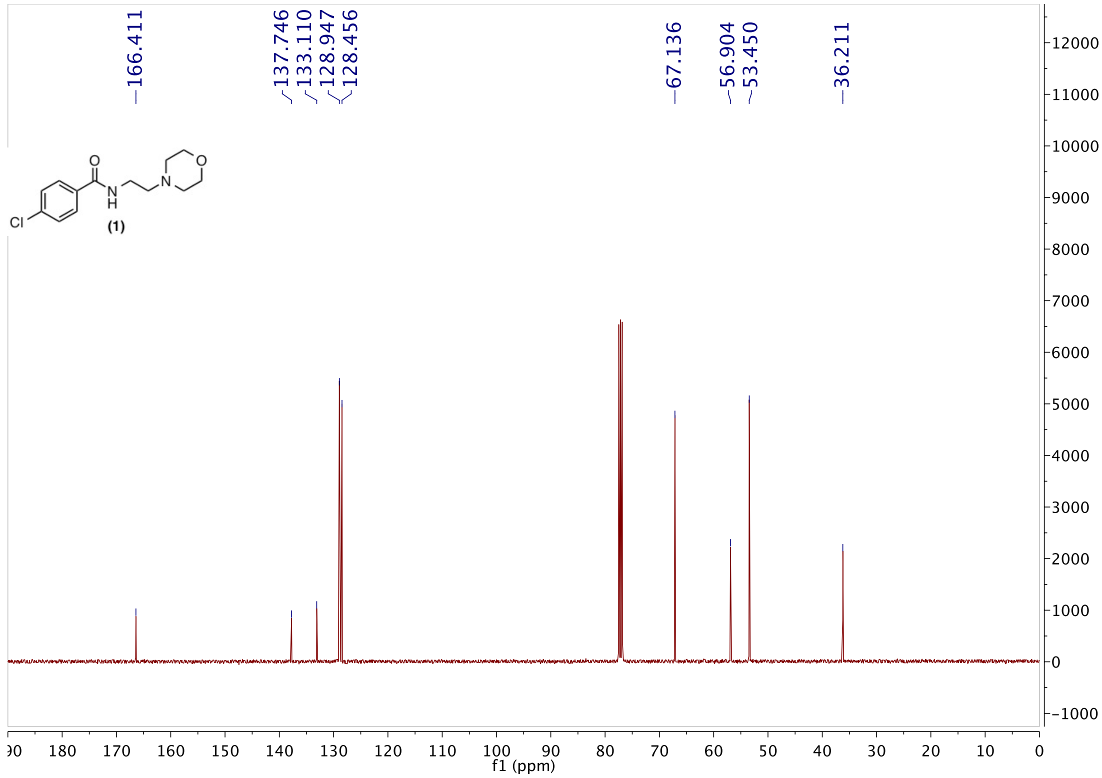

Supplementary Figure 1. ^{1}H NMR ($CDCl_3$, 400 MHz, panel a) and ^{13}C NMR ($CDCl_3$, 100MHz, panel b) of compound **1**.

a

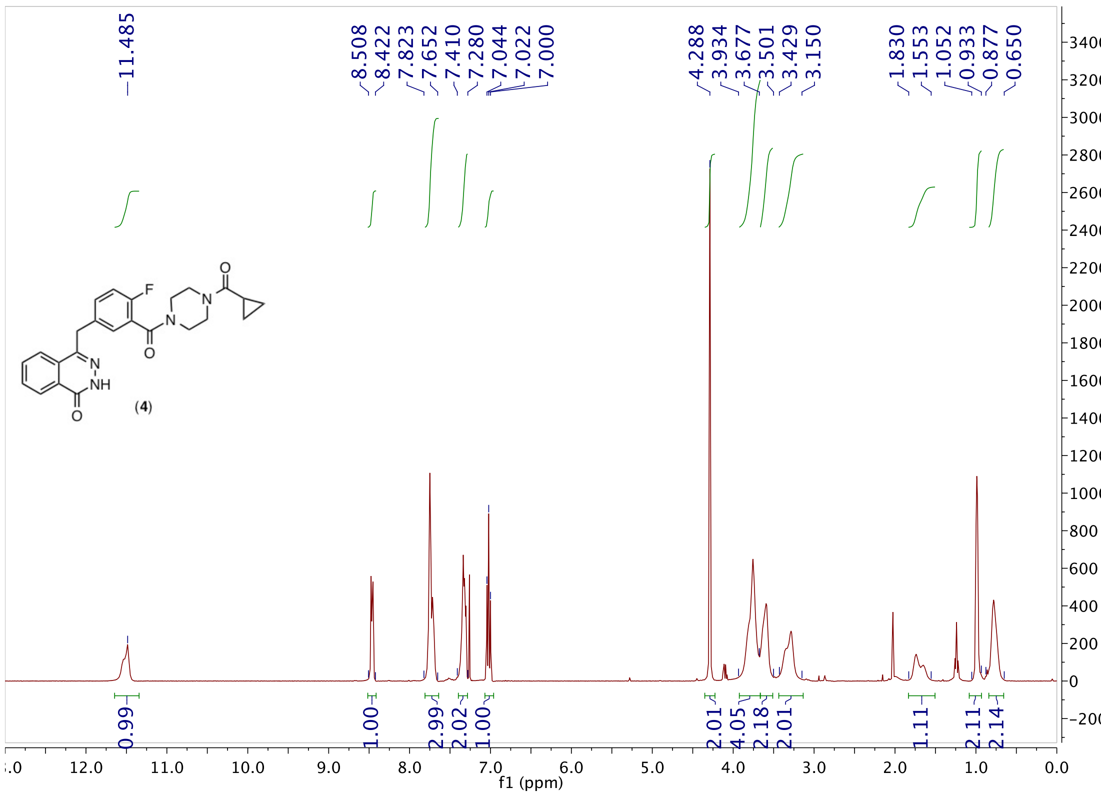

11.485
8.508
8.422
7.823
7.652
7.410
7.280
7.044
7.022
7.000
4.288
3.934
3.677
3.501
3.429
3.150
1.830
1.553
1.052
0.933
0.877
0.650
0.99
1.00
2.99
2.02
1.00
2.01
4.05
2.18
2.01
1.11
2.11
2.14
F
N
O
N
NH
(4)
f1 (ppm)

b

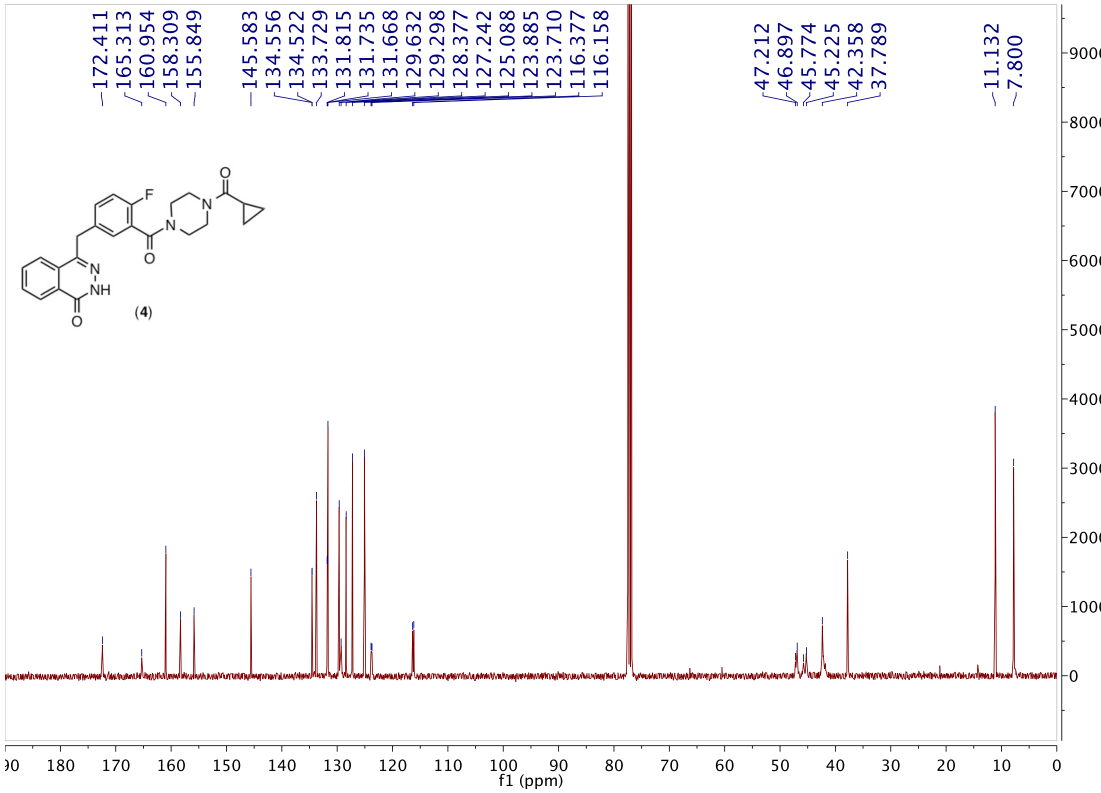

172.411
165.313
160.954
158.309
155.849
145.583
134.556
134.522
133.729
131.815
131.735
131.668
129.632
129.298
128.377
127.242
125.088
123.885
123.710
116.377
116.158
47.212
46.897
45.774
45.225
42.358
37.789
11.132
7.800
F
N
O
N
NH
(4)
f1 (ppm)

c

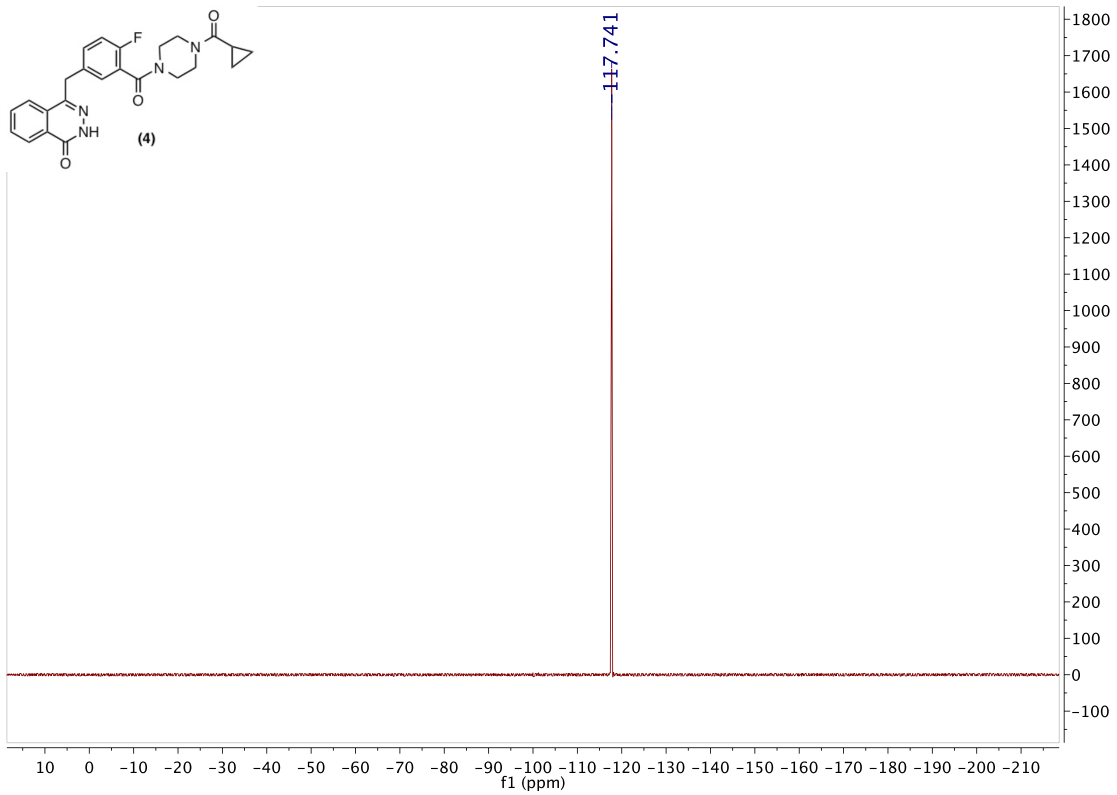

Supplementary Figure 2. ^{1}H NMR ($CDCl_3$, 400 MHz, panel a), ^{13}C NMR ($CDCl_3$, 100 MHz, panel b) and ^{19}F NMR ($CDCl_3$, 367 MHz, panel c) of compound **4**.

a

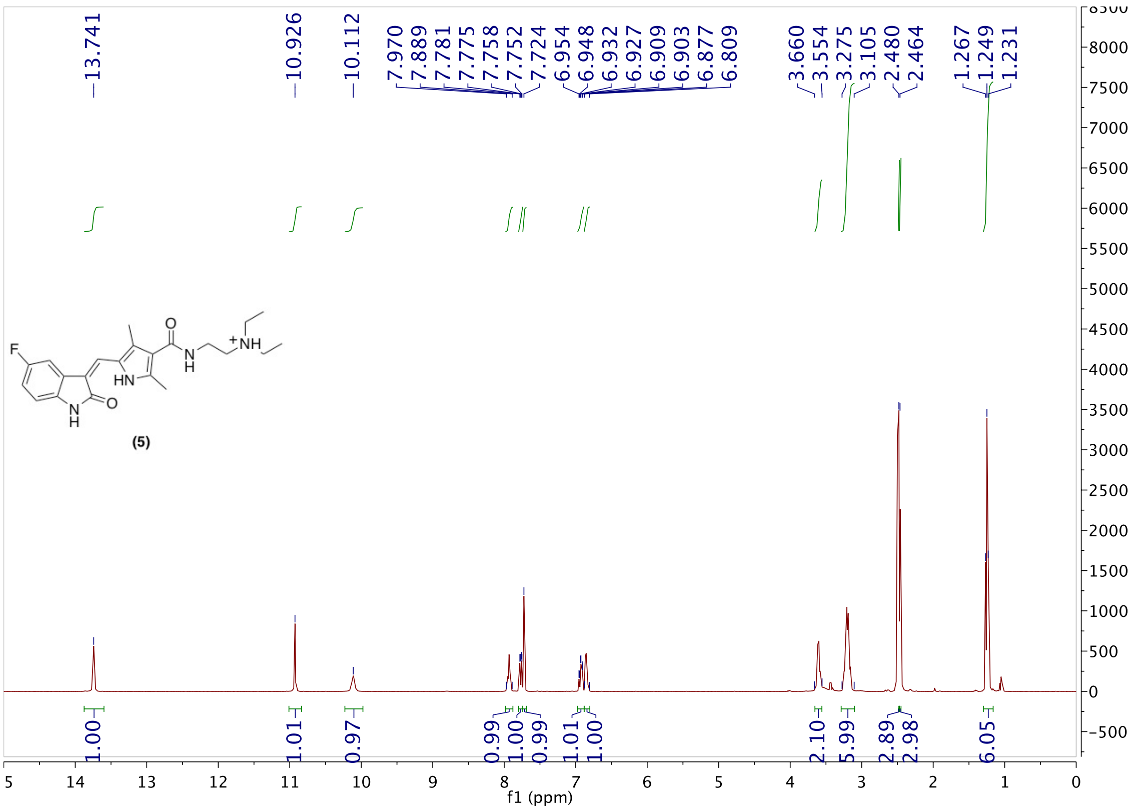

13.741
10.926
10.112
7.970
7.889
7.781
7.775
7.758
7.752
7.724
6.954
6.948
6.932
6.927
6.909
6.903
6.877
6.809
3.660
3.554
3.275
3.105
2.480
2.464
1.267
1.249
1.231
F
O
HN
N
H
+NH
H
O
(5)
1.00
1.01
0.97
0.99
1.00
0.99
1.01
1.00
2.10
5.99
2.89
2.98
6.05
f1 (ppm)

b

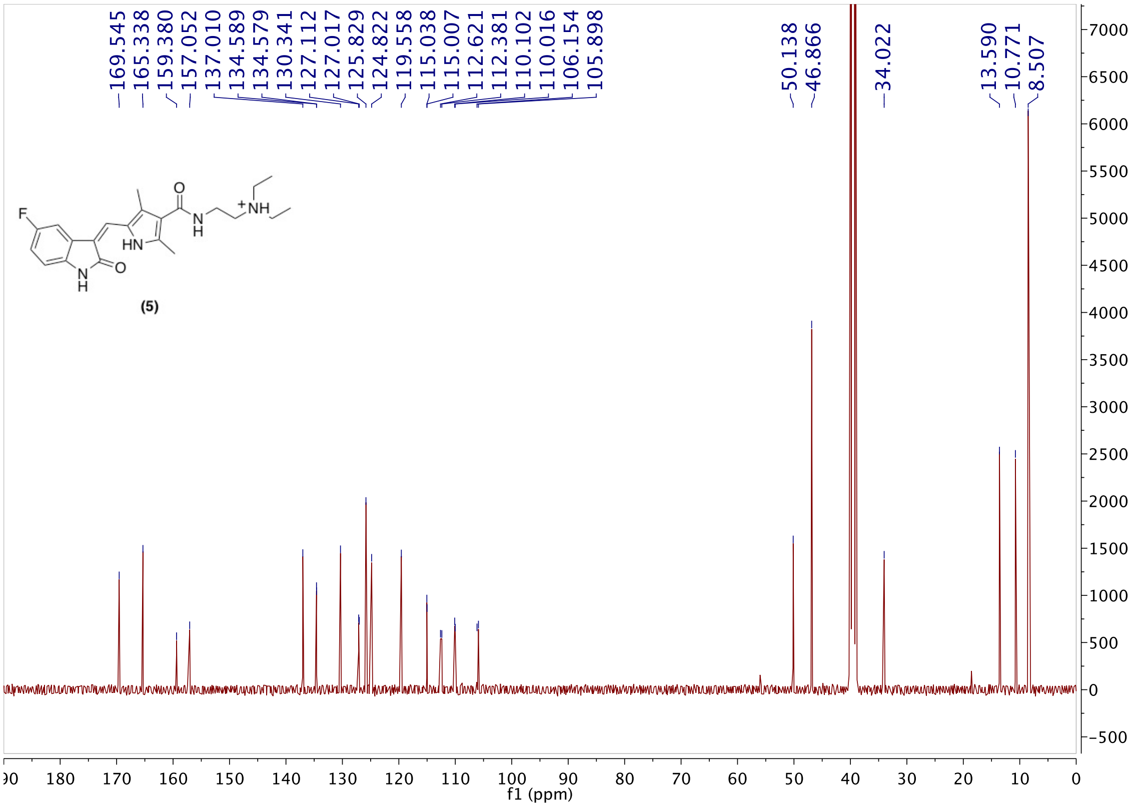

169.545
165.338
159.380
157.052
137.010
134.589
134.579
130.341
127.112
127.017
125.829
124.822
119.558
115.038
115.007
112.621
112.381
110.102
110.016
106.154
105.898
50.138
46.866
34.022
13.590
10.771
8.507
F
O
HN
N
H
+NH
H
O
(5)
f1 (ppm)

c

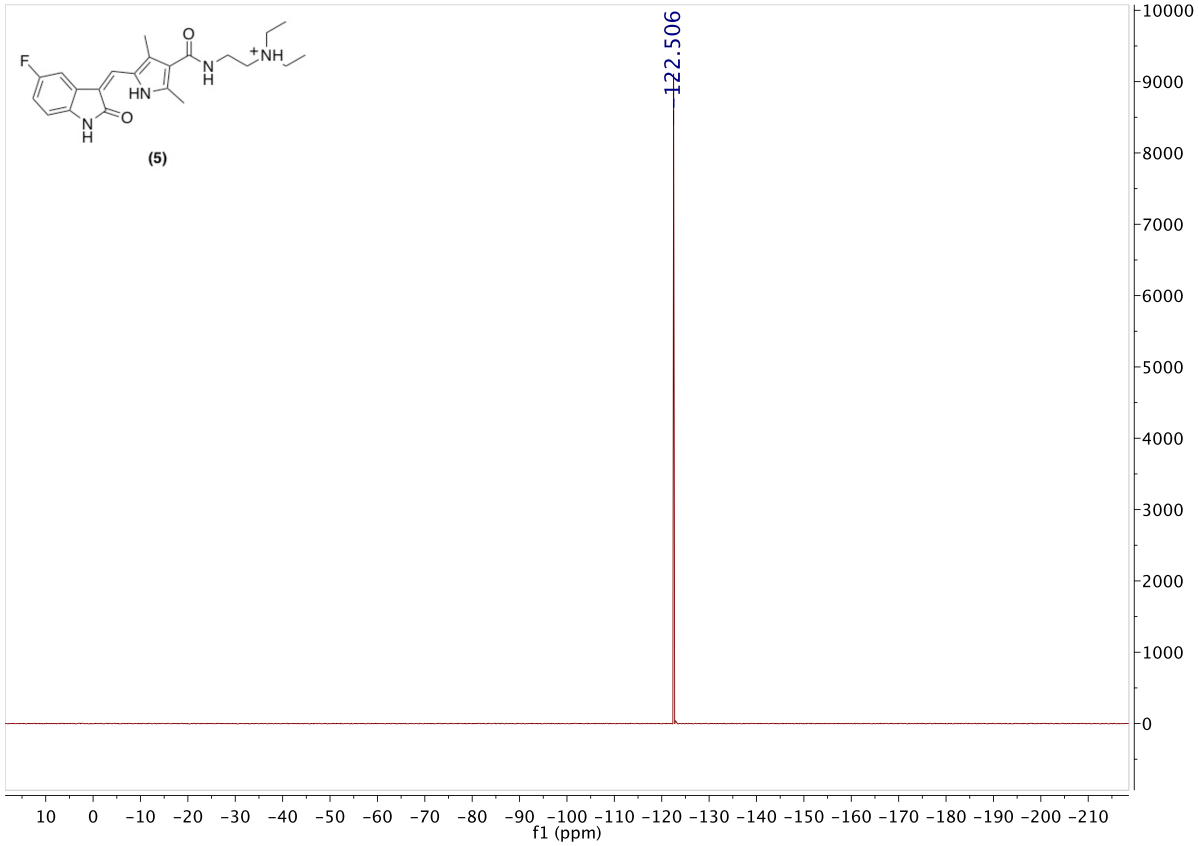

Supplementary Figure 3. ^{1}H NMR (DMSO-d_6, 400 MHz, panel a), ^{13}C NMR (DMSO-d_6, 100 MHz, panel b) and ^{19}F NMR (DMSO-d_6, 367 MHz, panel c) of compound **5**.

a

b

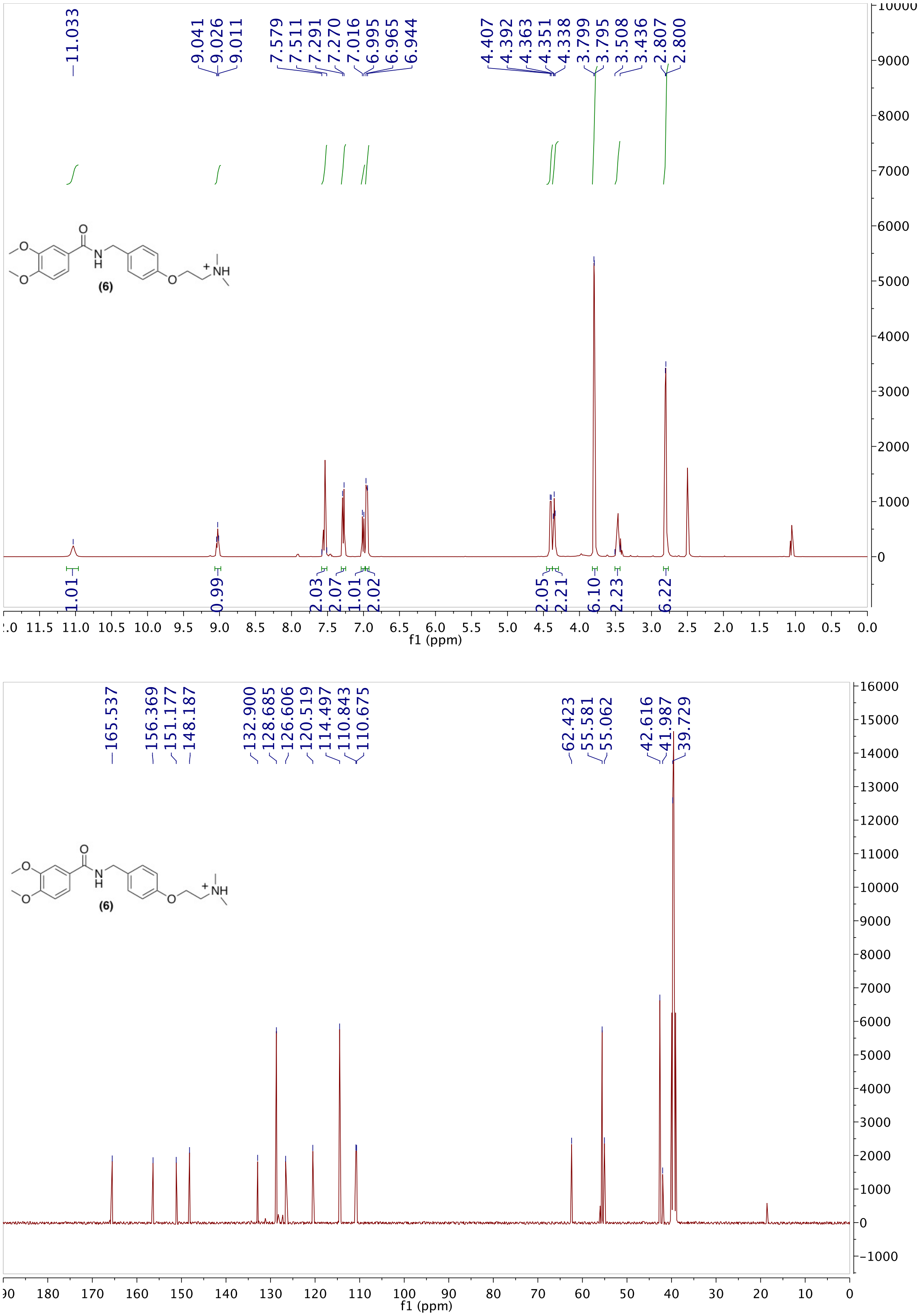

Supplementary Figure 4. ^{1}H NMR (DMSO-d_6, 400 MHz, panel a) and ^{13}C NMR (DMSO-d_6, 100 MHz, panel b) of compound **6**.

a

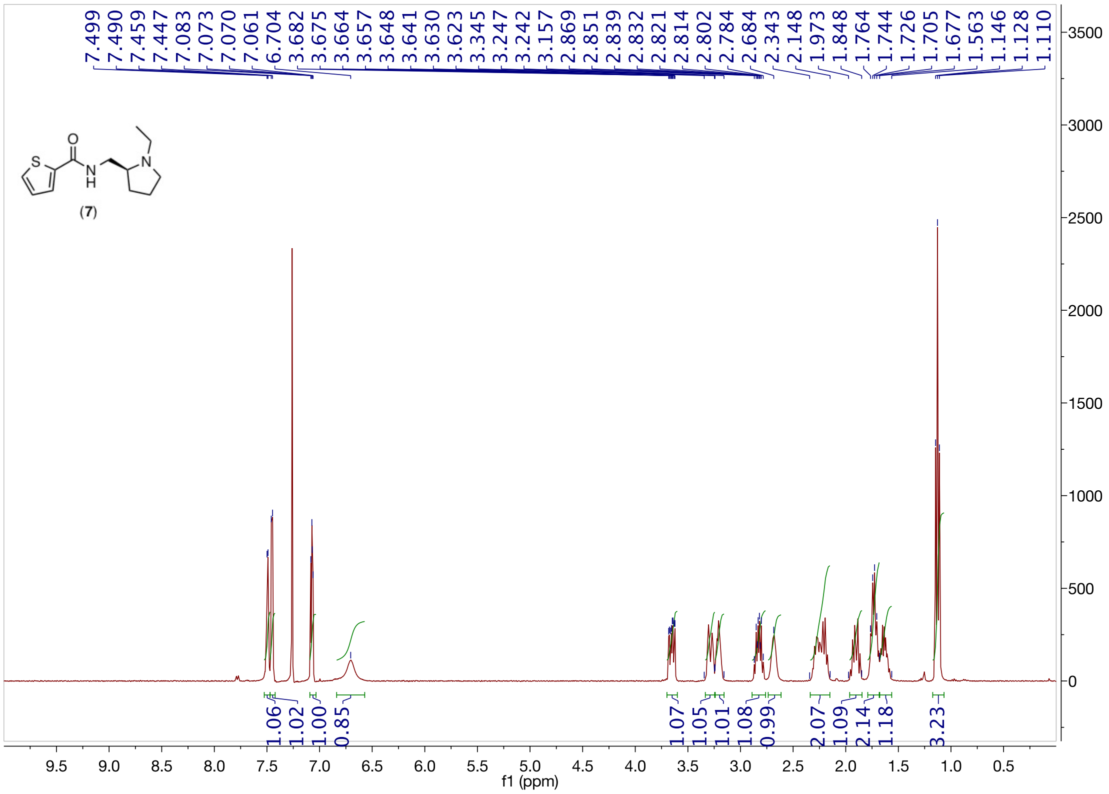

b

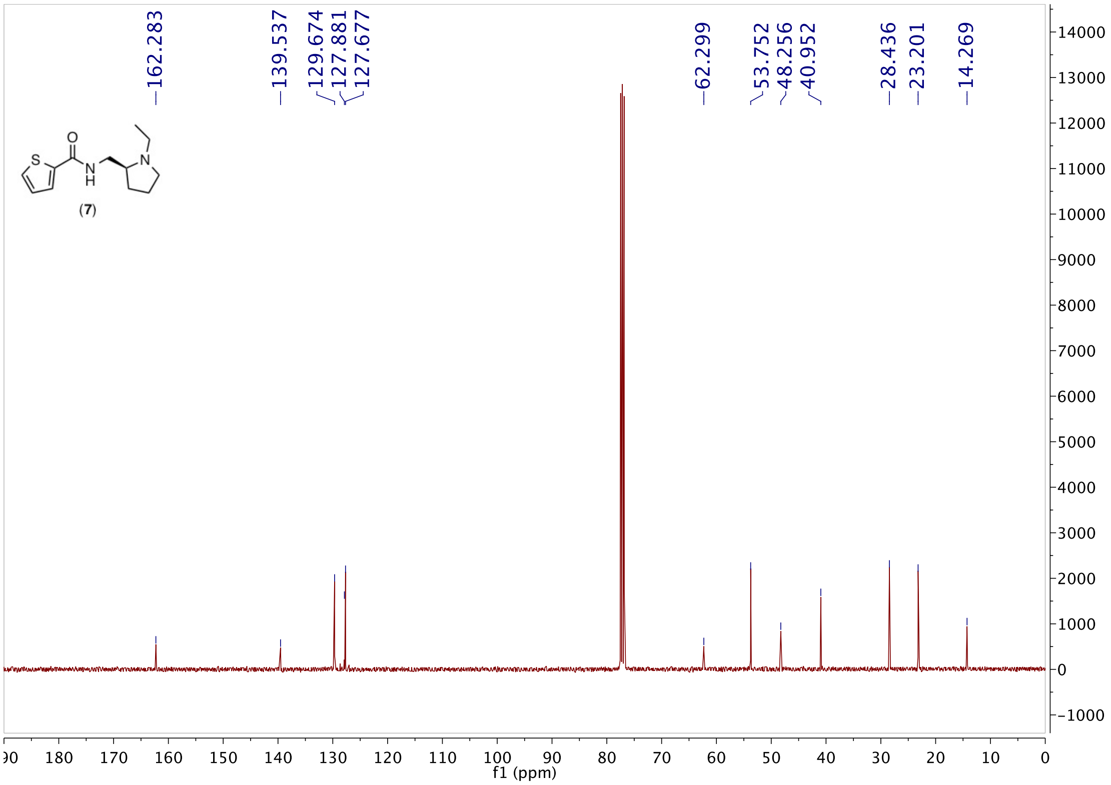

Supplementary Figure 5. ^{1}H NMR ($CDCl_3$, 400 MHz, panel a) and ^{13}C NMR ($CDCl_3$, 100 MHz, panel b) of compound **7**.

a

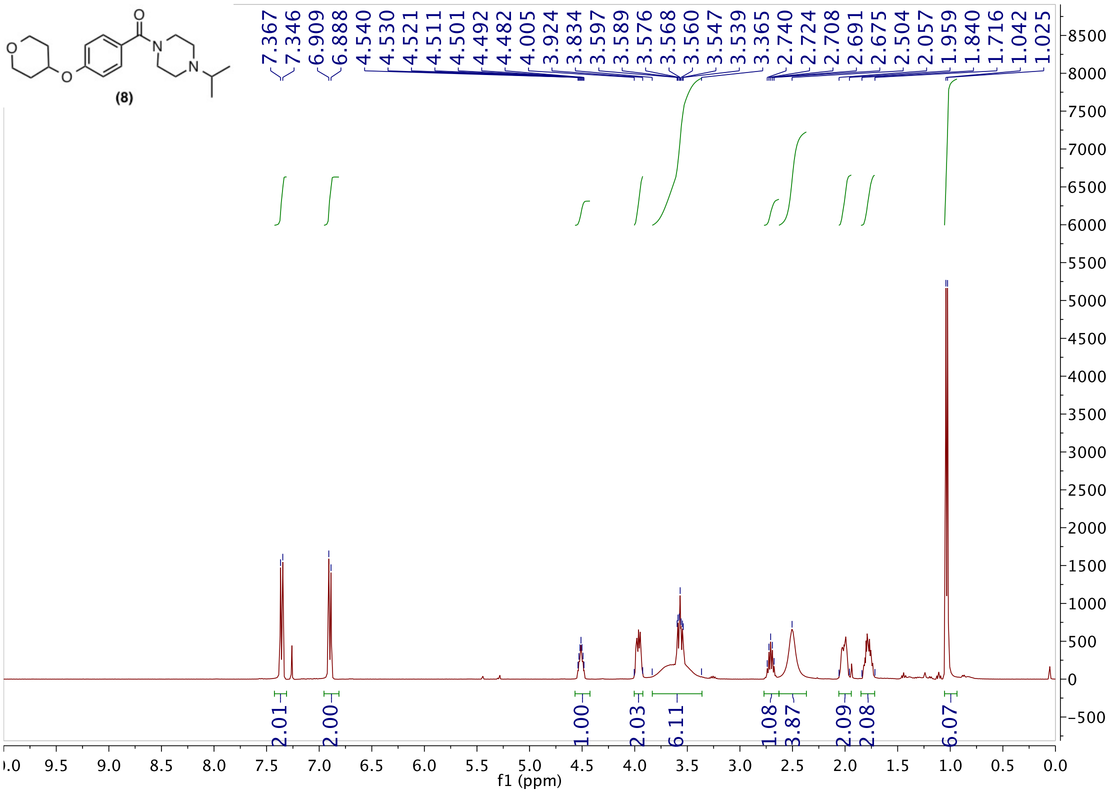

b

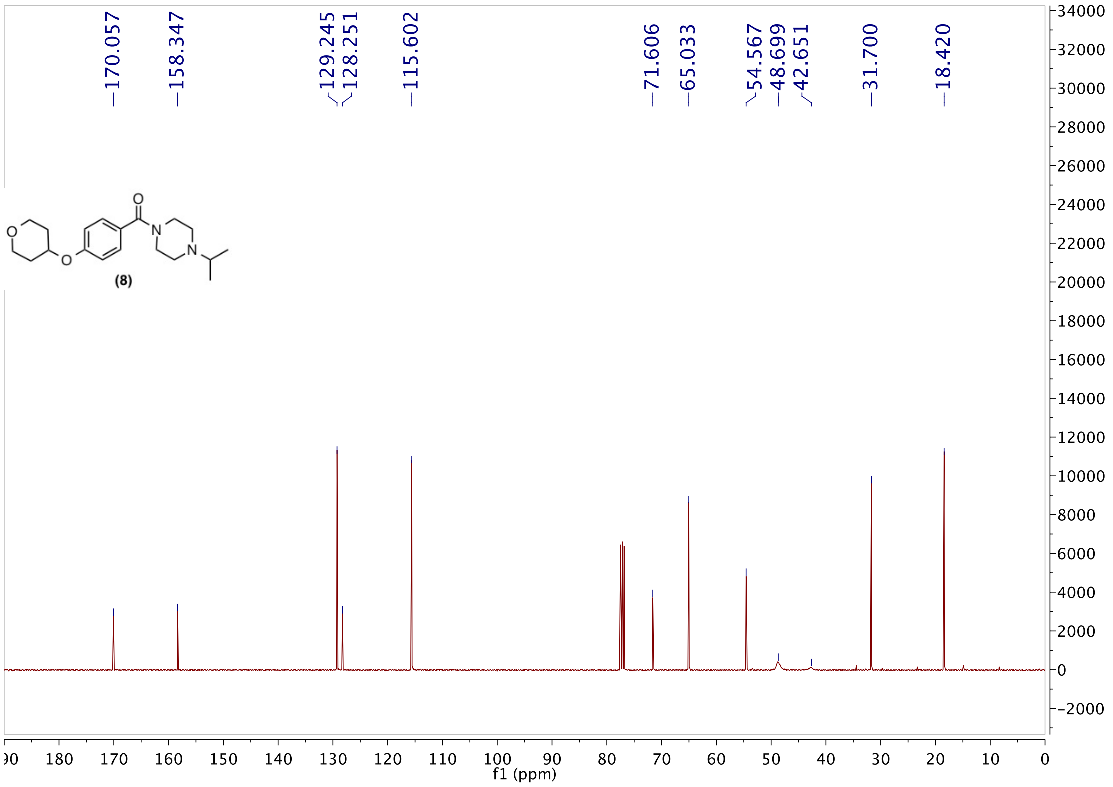

Supplementary Figure 6. ^{1}H NMR ($CDCl_3$, 400 MHz, panel a) and ^{13}C NMR ($CDCl_3$, 100 MHz, panel b) of compound **8**.

a

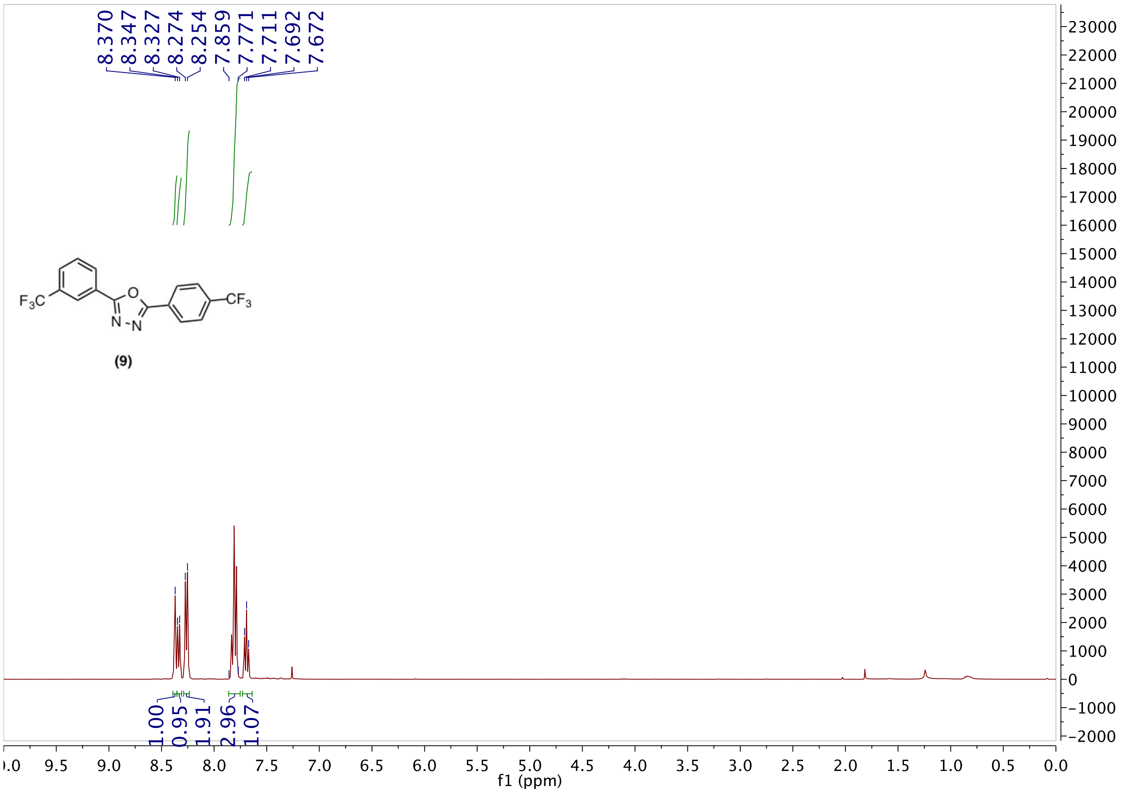
8.370
8.347
8.327
8.274
8.254
7.859
7.771
7.711
7.692
7.672
F3C
O
N-N
CF3
(9)
1.00
0.95
1.91
2.96
1.07
f1 (ppm)

b

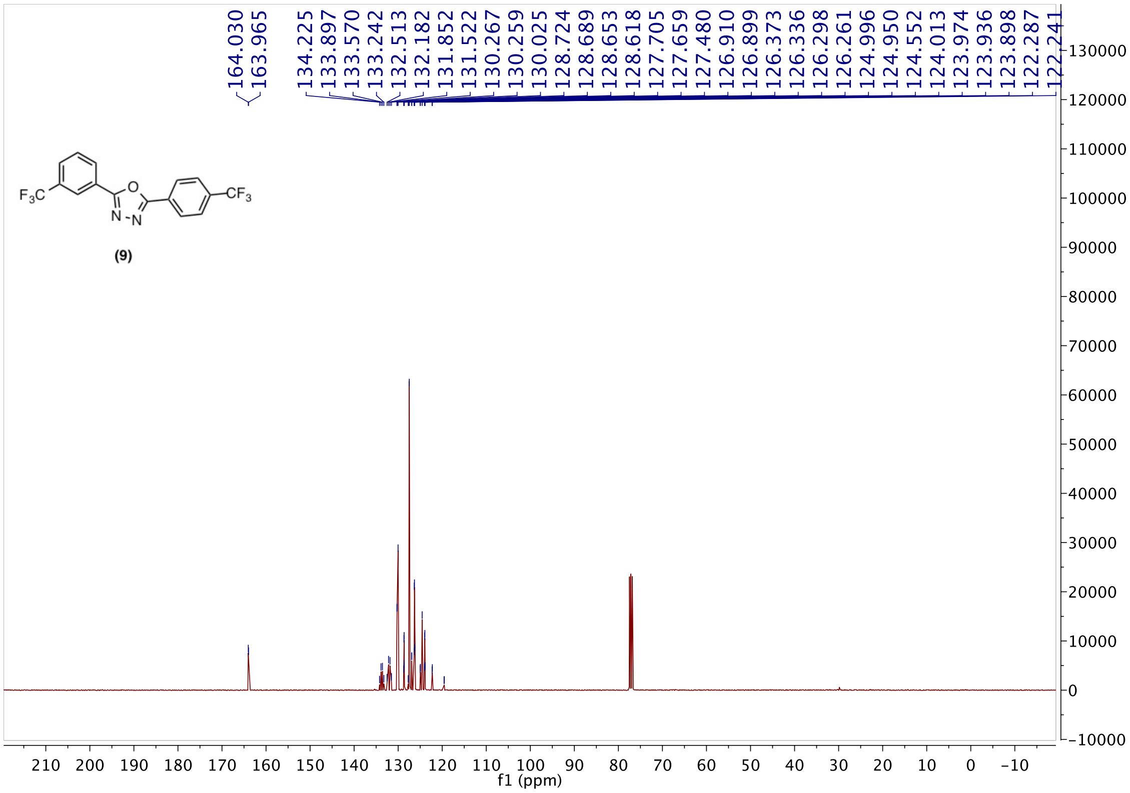
164.030
163.965
134.225
133.897
133.570
133.242
132.513
132.182
131.852
131.522
130.267
130.259
130.025
128.724
128.689
128.653
128.618
127.705
127.659
127.480
126.910
126.899
126.373
126.336
126.298
126.261
124.996
124.950
124.552
124.013
123.974
123.936
123.898
122.287
122.241
F3C
O
N-N
CF3
(9)
f1 (ppm)

c

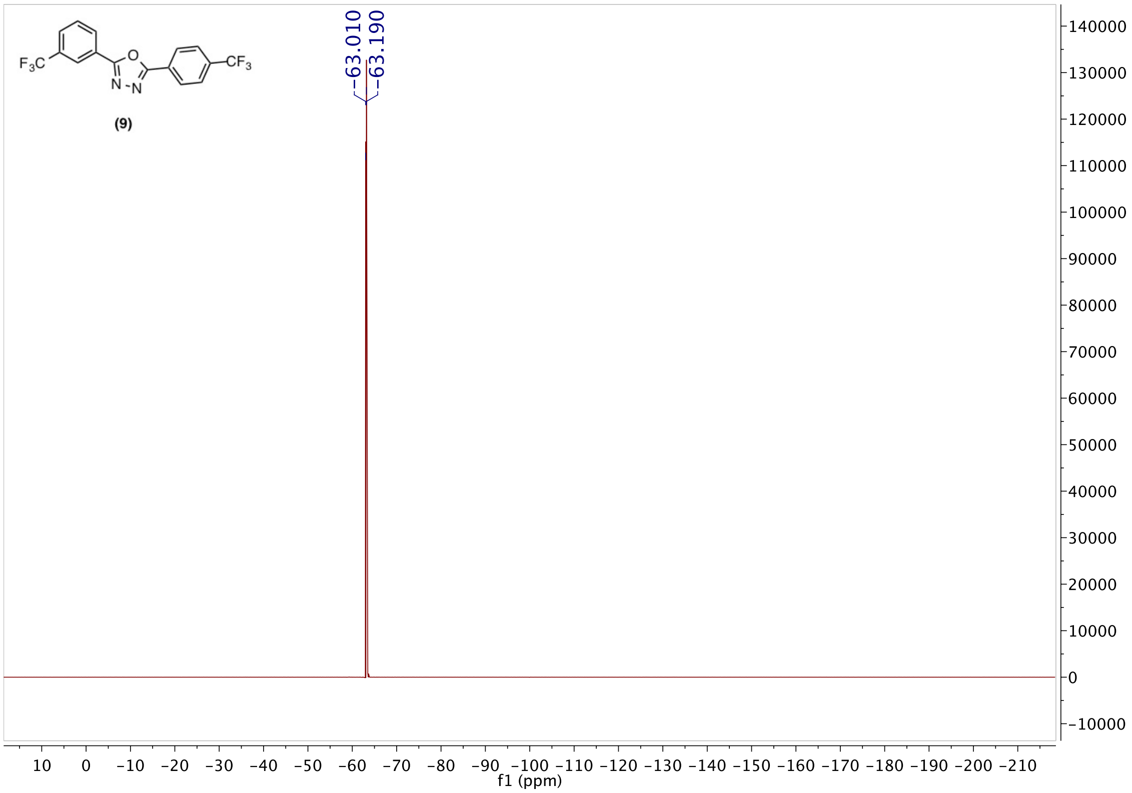

Supplementary Figure 7. ^{1}H NMR ($CDCl_3$, 400 MHz, panel a), ^{13}C NMR ($CDCl_3$, 100 MHz, panel b) and ^{19}F NMR ($CDCl_3$, 367 MHz, panel c) of compound **9**.

a

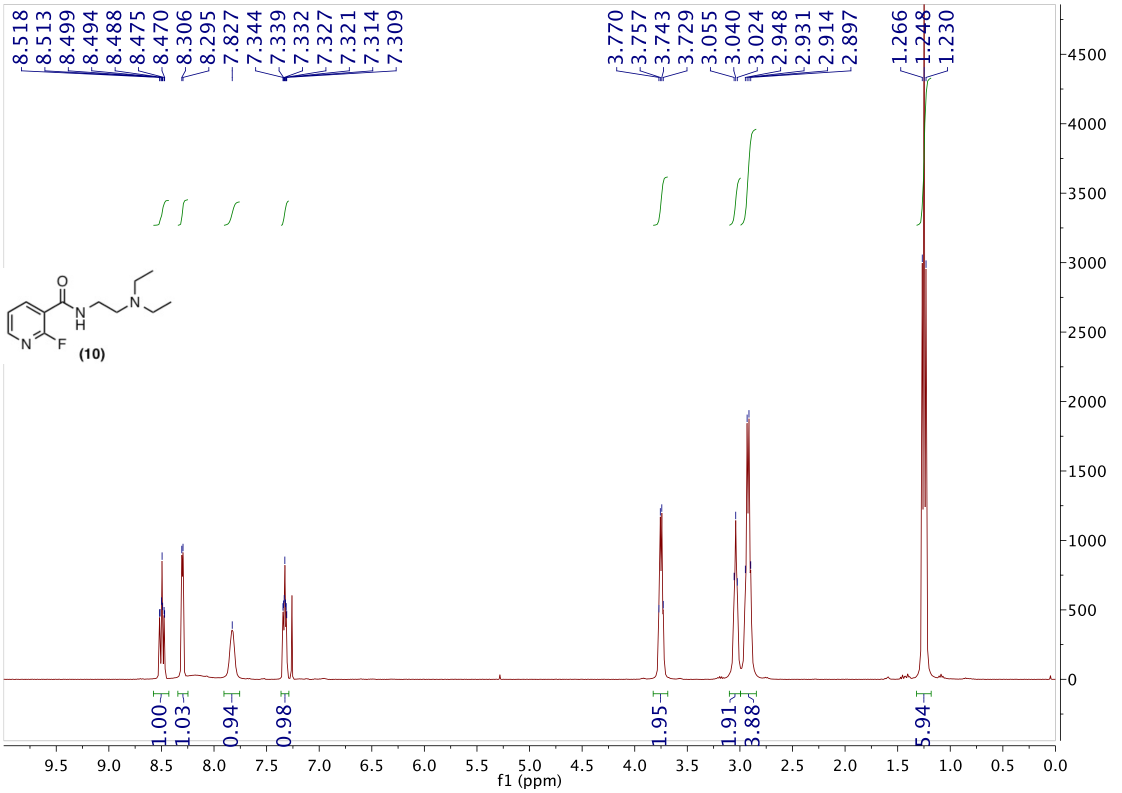

8.518
8.513
8.499
8.494
8.488
8.475
8.470
8.306
8.295
7.827
7.344
7.339
7.332
7.327
7.321
7.314
7.309
3.770
3.757
3.743
3.729
3.055
3.040
3.024
2.948
2.931
2.914
2.897
1.266
1.248
1.230
O
N
H
N
N
F
(10)
1.00
1.03
0.94
0.98
1.95
1.91
3.88
5.94
f1 (ppm)

b

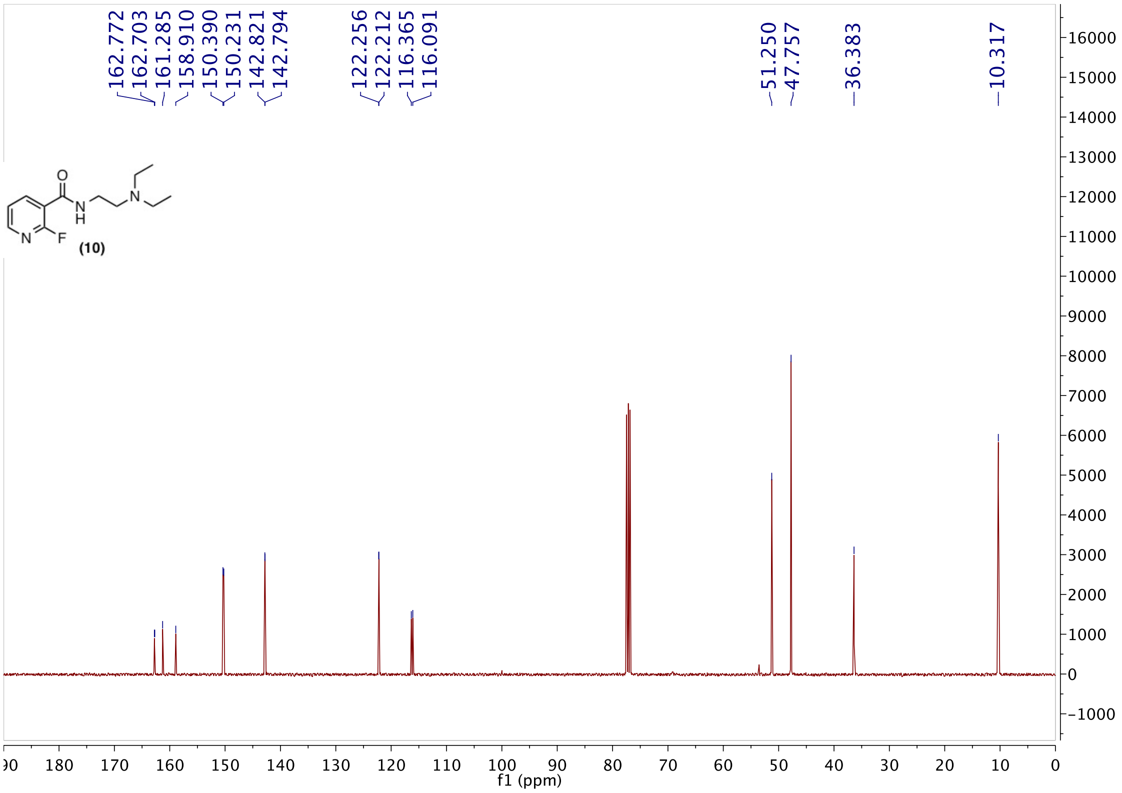

162.772
162.703
161.285
158.910
150.390
150.231
142.821
142.794
122.256
122.212
116.365
116.091
51.250
47.757
36.383
10.317
O
N
H
N
N
F
(10)
f1 (ppm)

C

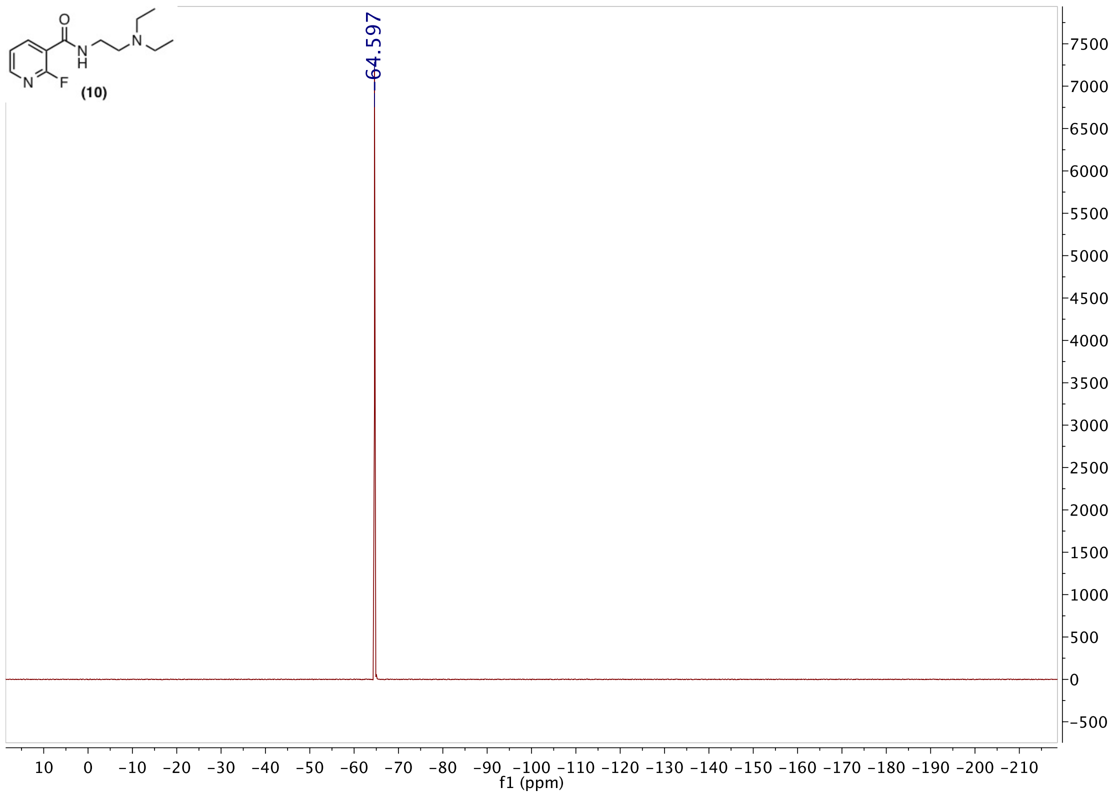

Supplementary Figure 8. ^{1}H NMR ($CDCl_3$, 400 MHz, panel a), ^{13}C NMR ($CDCl_3$, 100 MHz, panel b) and ^{19}F NMR ($CDCl_3$, 367 MHz, panel c) of compound **10**.

a

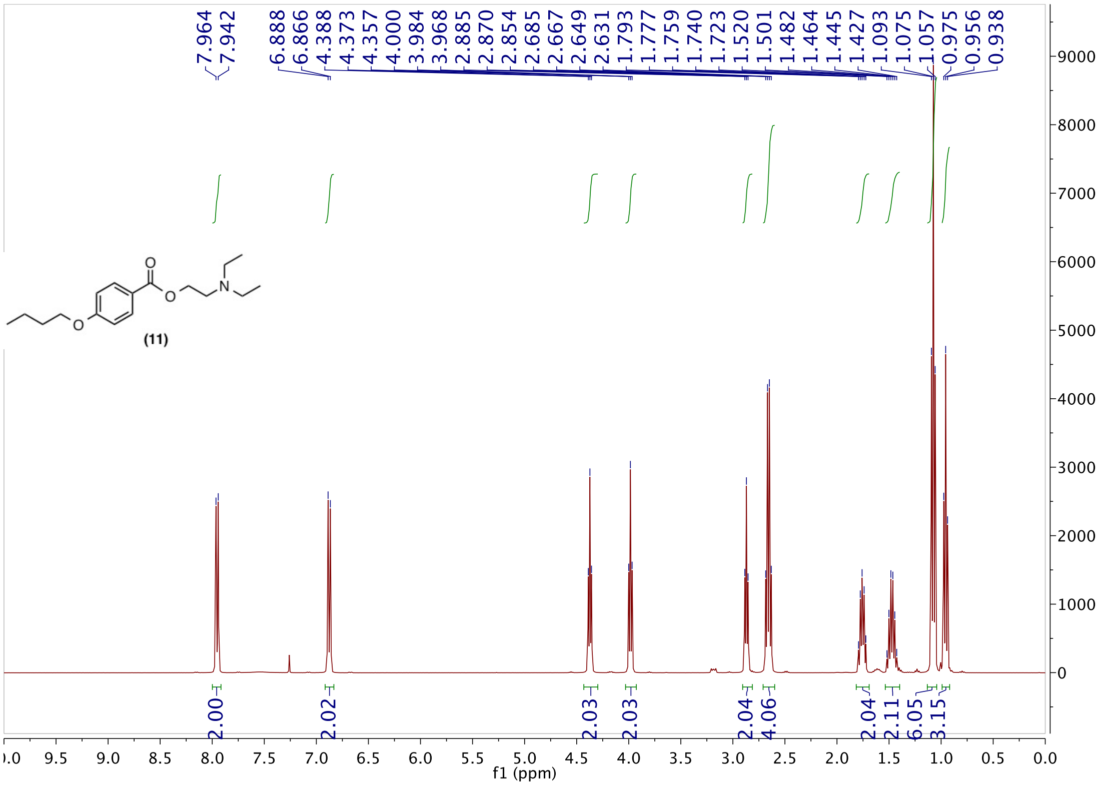

b

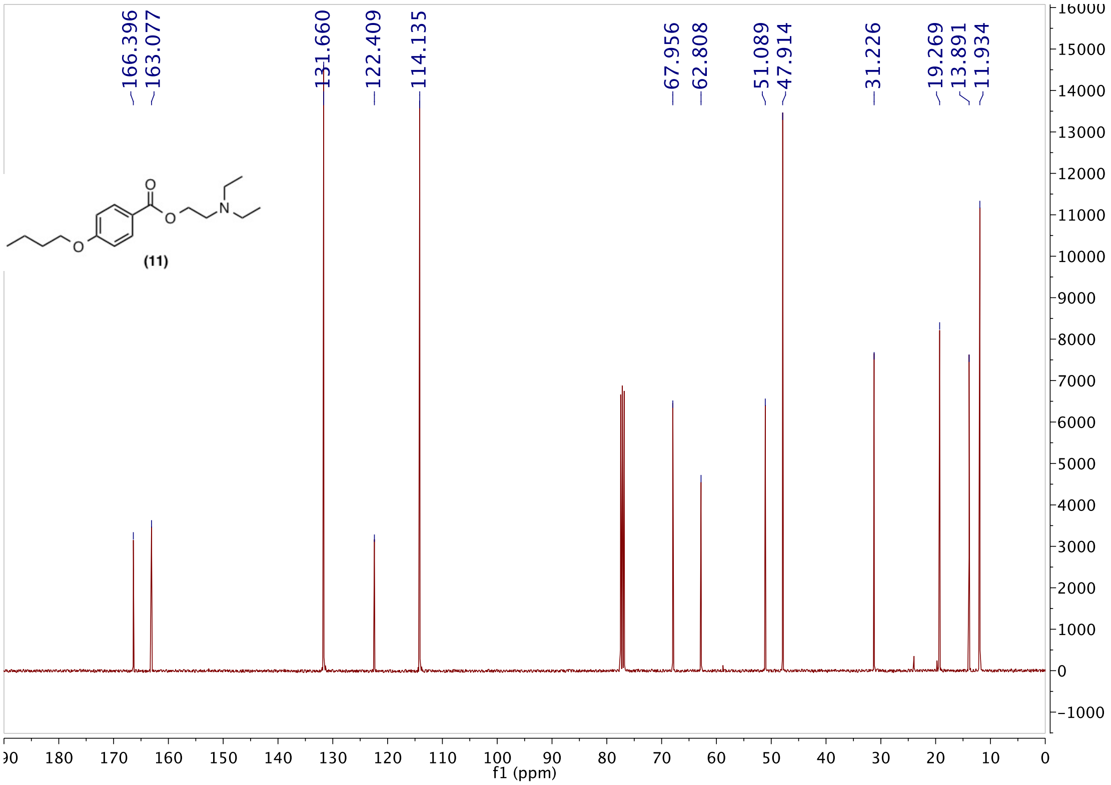

Supplementary Figure 9. ^{1}H NMR ($CDCl_3$, 400 MHz, panel a) and ^{13}C NMR ($CDCl_3$, 100 MHz, panel b) of compound **11**.

a

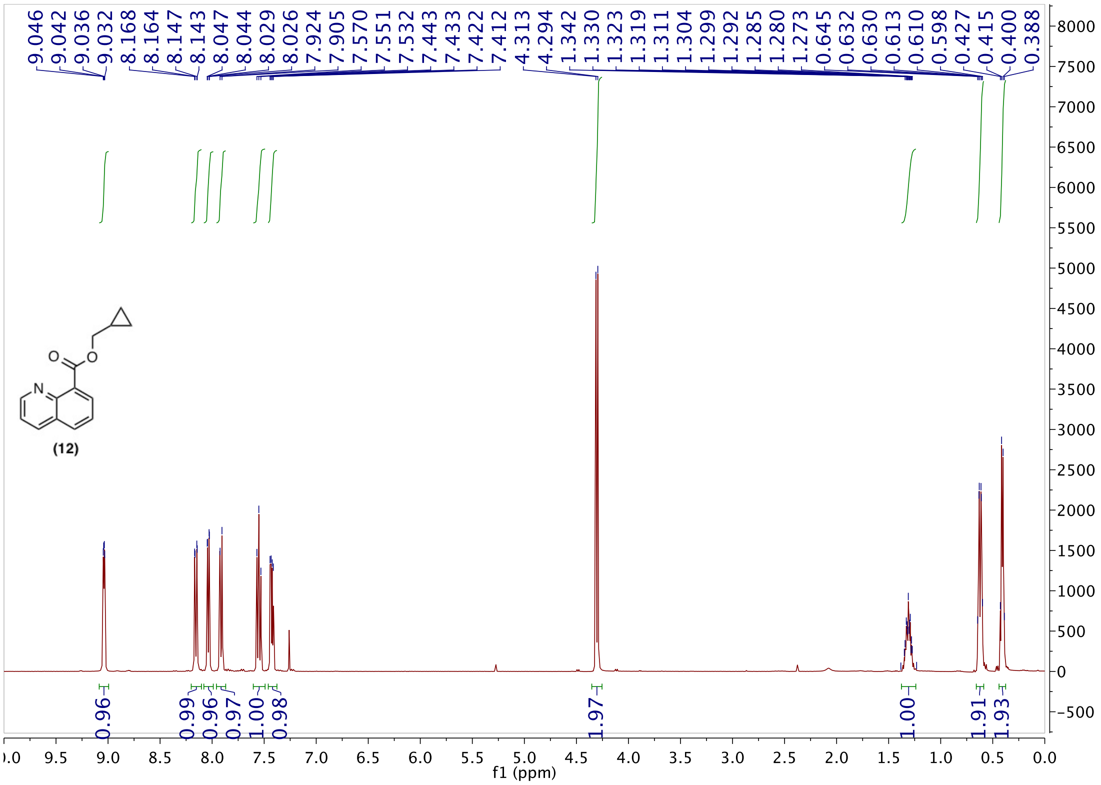

b

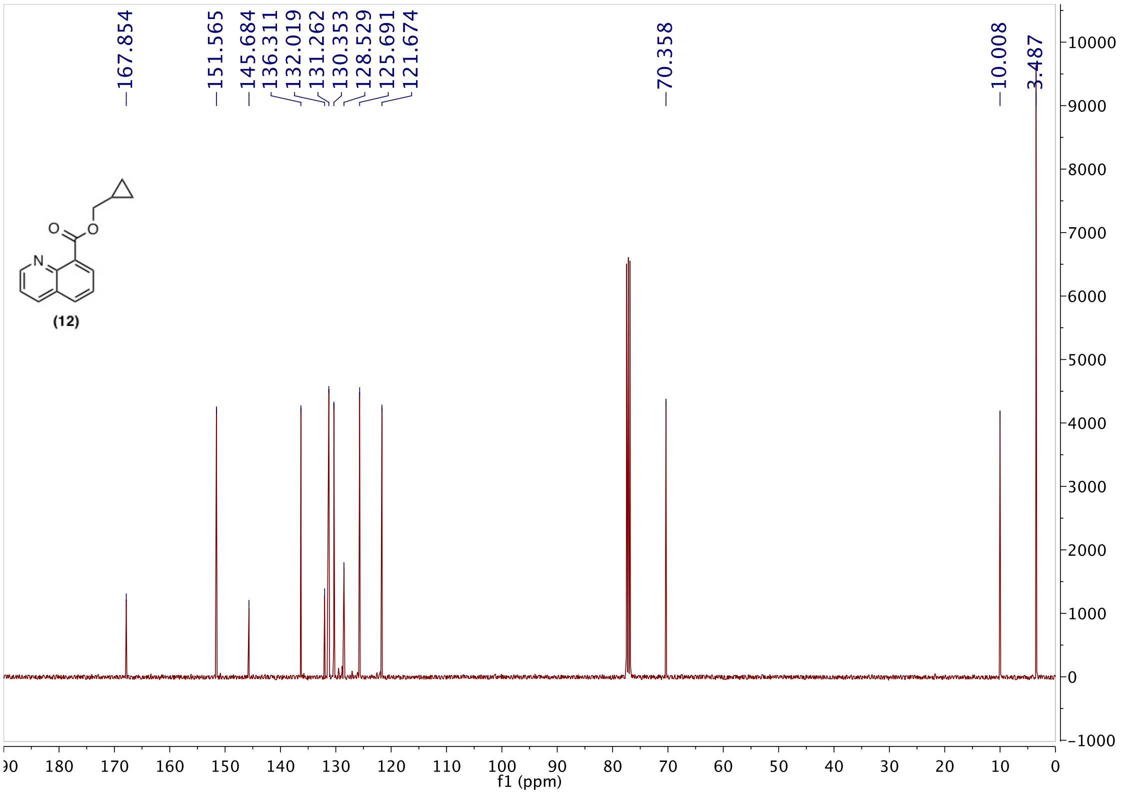

Supplementary Figure 10. ^{1}H NMR ($CDCl_3$, 400 MHz, panel a) and ^{13}C NMR ($CDCl_3$, 100 MHz, panel b) of compound (**12**).

a

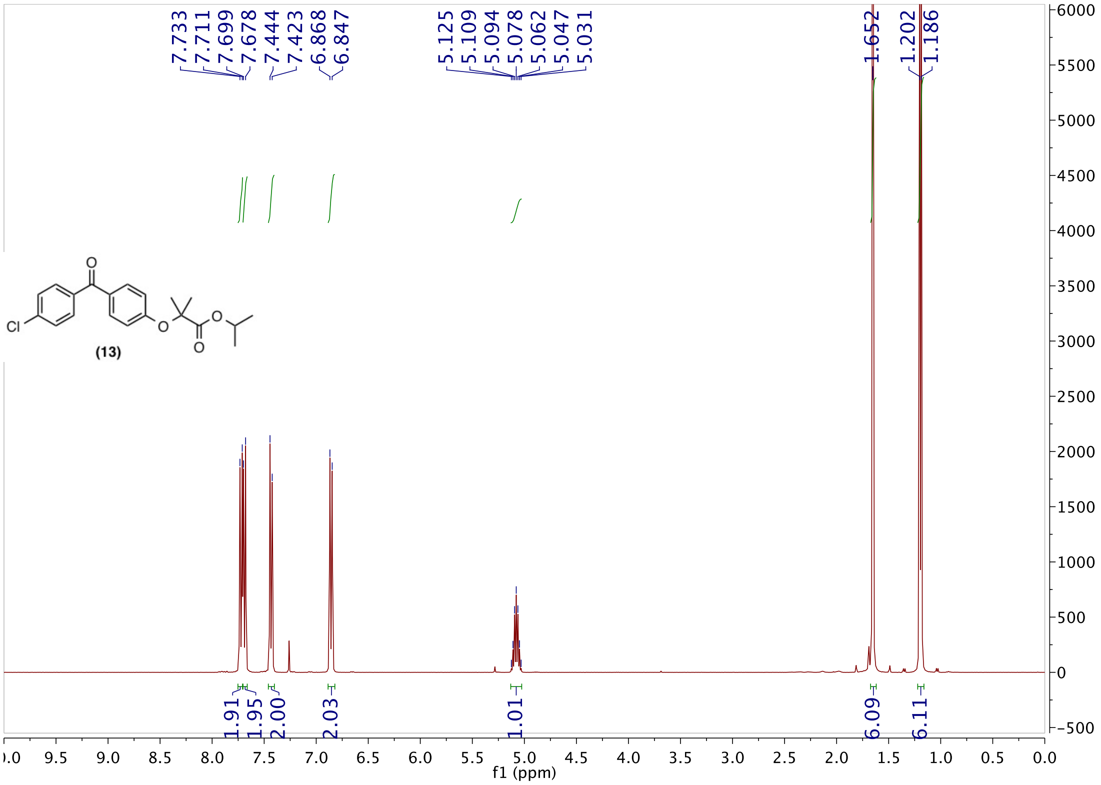

b

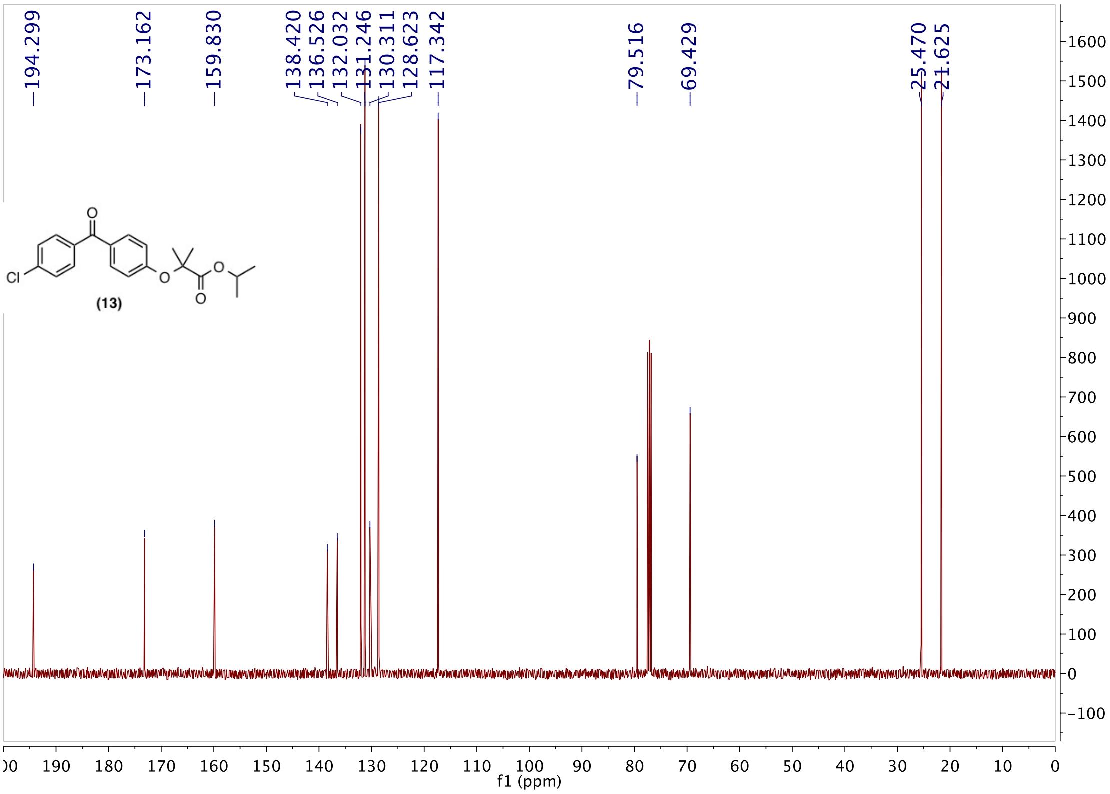

Supplementary Figure 11. ^{1}H NMR ($CDCl_3$, 400 MHz, panel a) and ^{13}C NMR ($CDCl_3$, 100 MHz, panel b) of compound **13**.

a

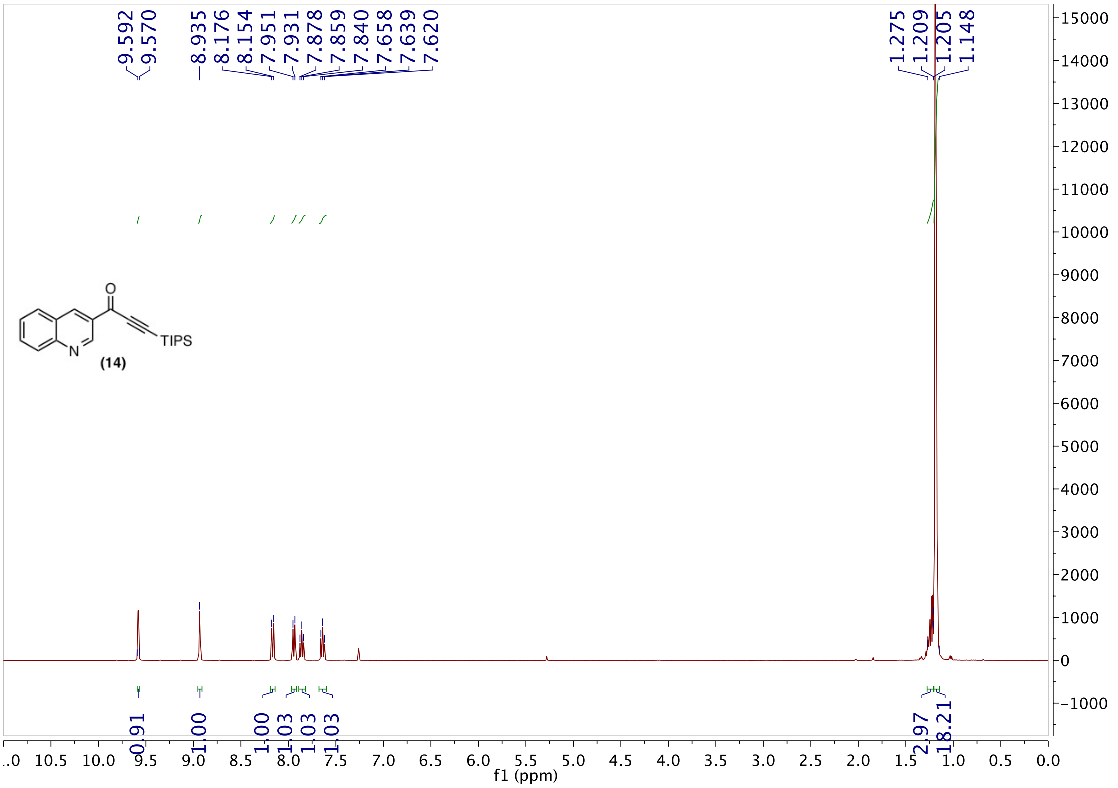

b

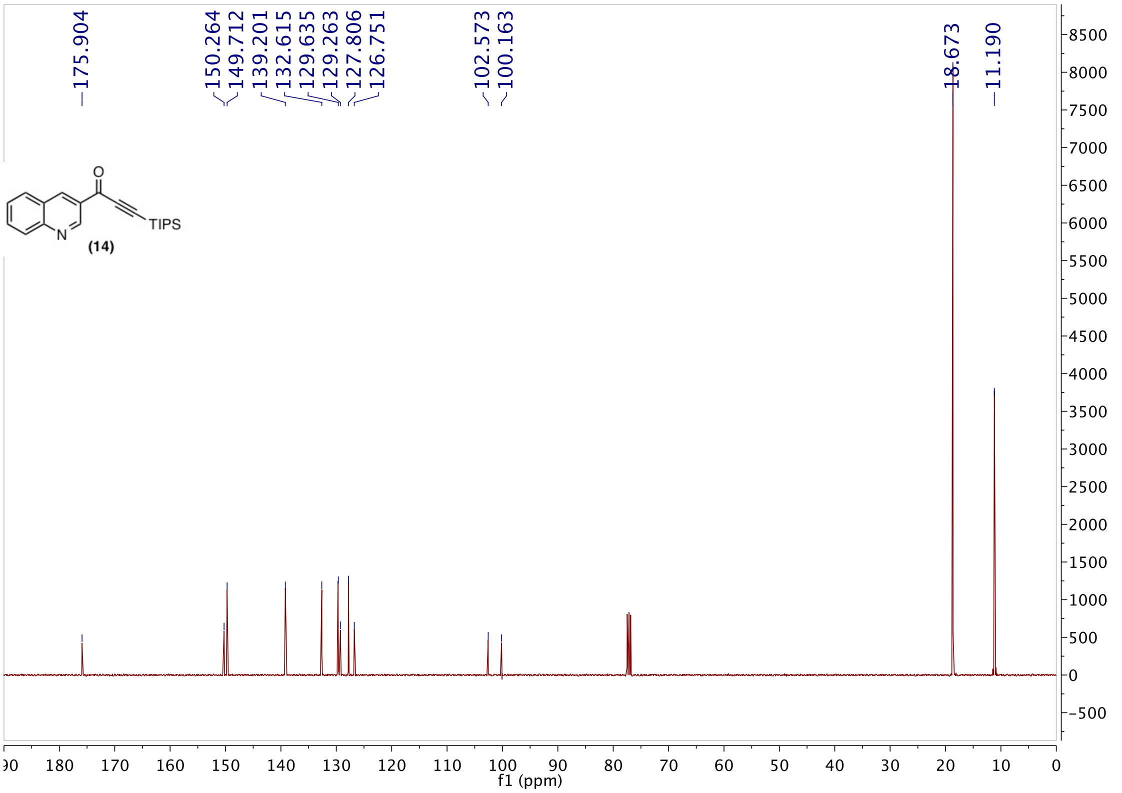

Supplementary Figure 12. ^{1}H NMR ($CDCl_3$, 400 MHz, panel a) and ^{13}C NMR ($CDCl_3$, 100 MHz, panel b) of compound **14**.

a

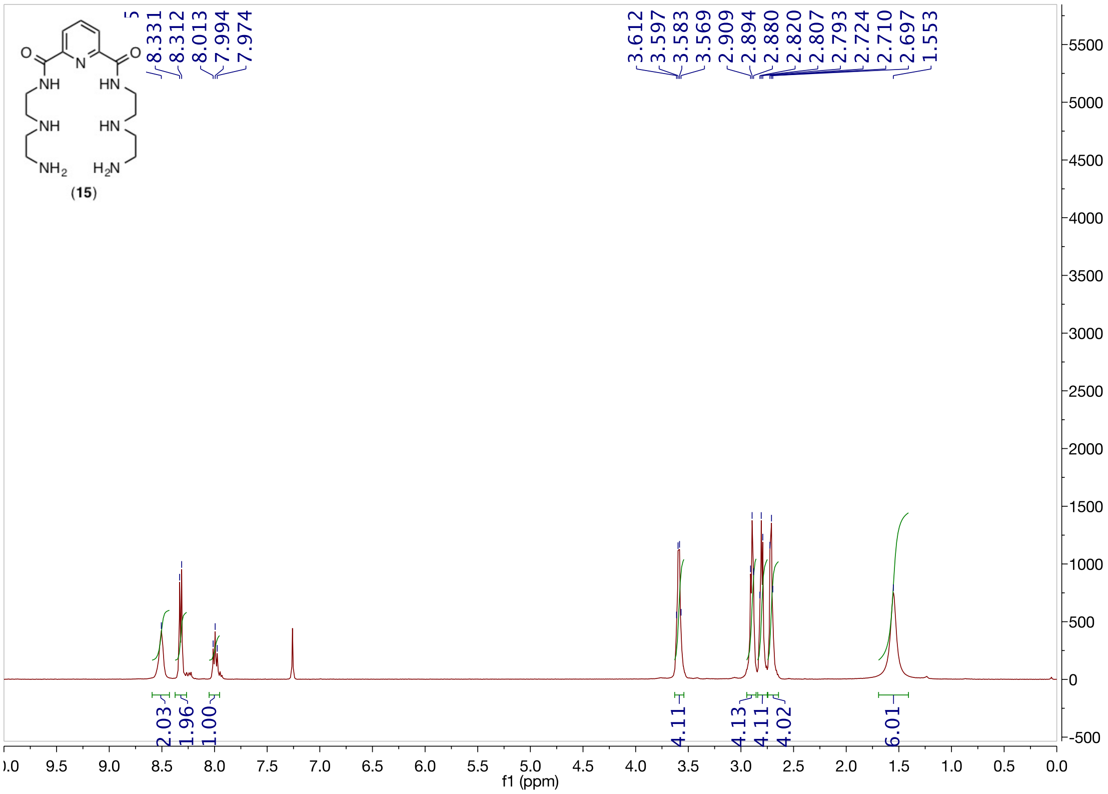

b

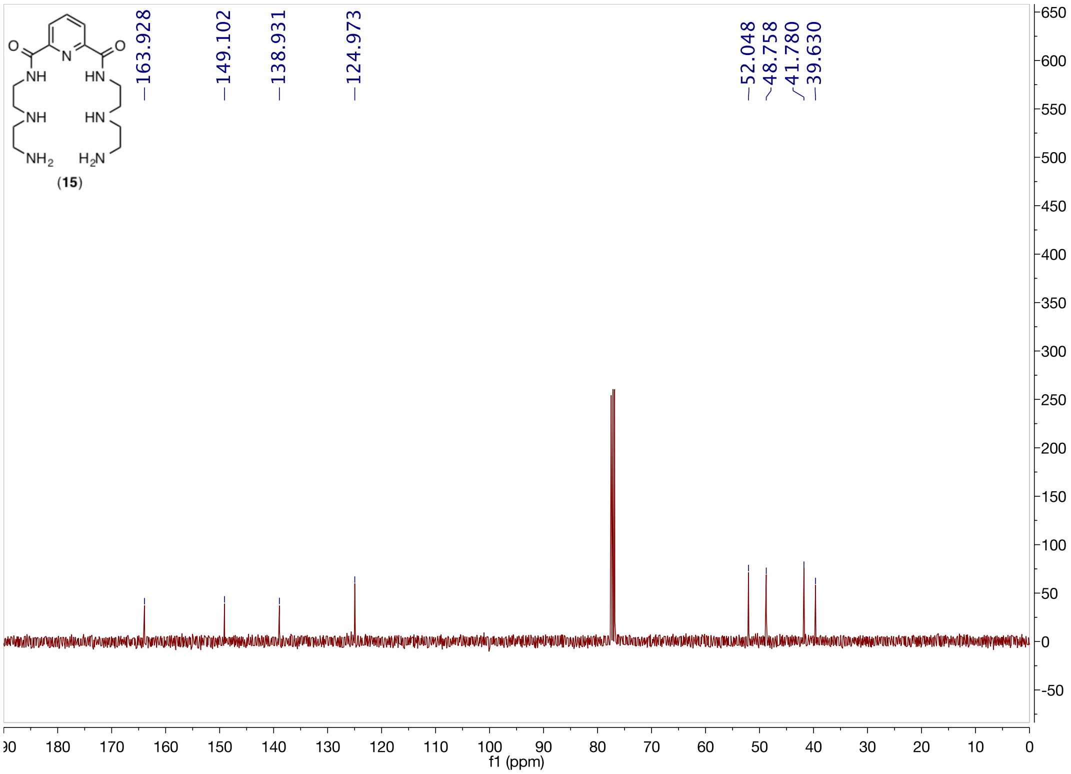

Supplementary Figure 13. ^{1}H NMR ($CDCl_3$, 400 MHz, panel a) and ^{13}C NMR ($CDCl_3$, 100 MHz, panel b) of compound **15**.

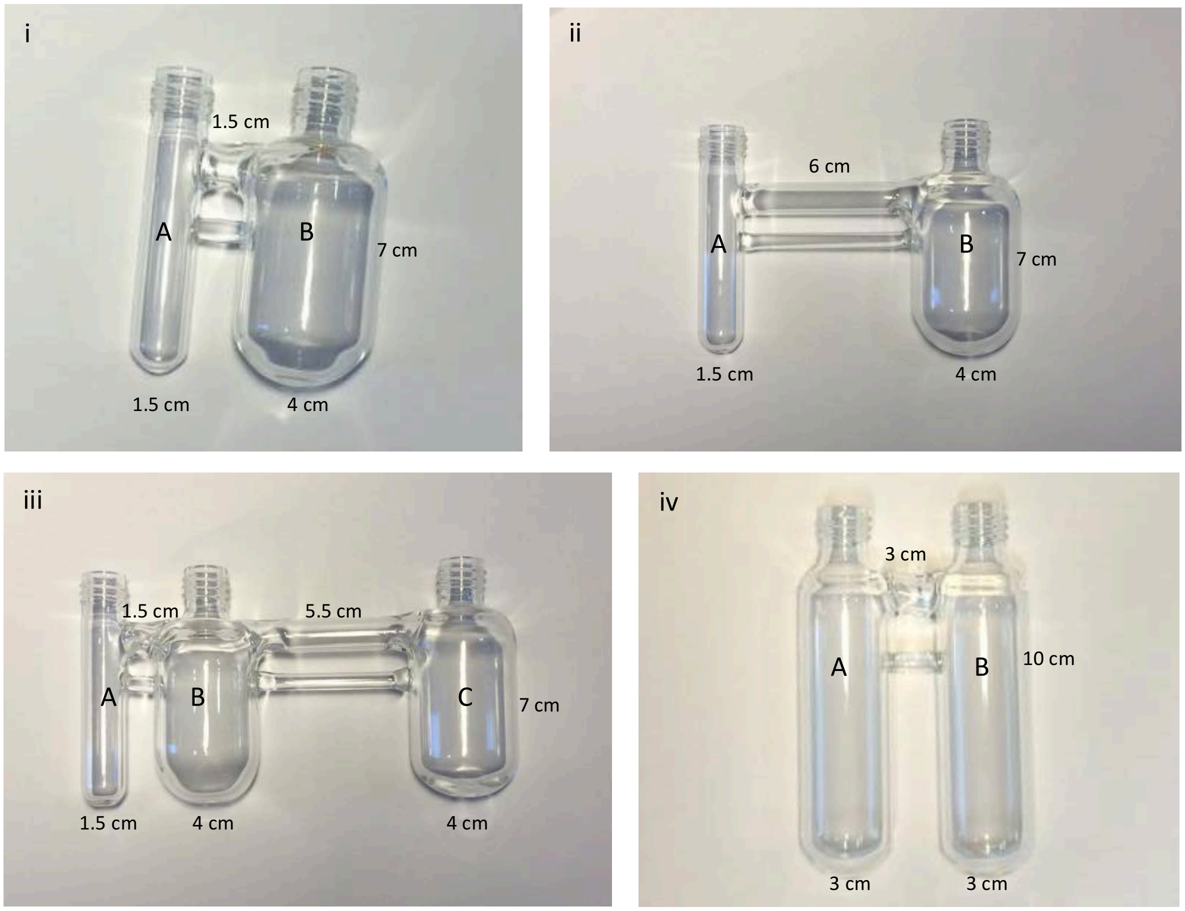

Supplementary Figure 14. Various types of glassware. Two or three vials of the same or different sizes are connected with glass bridges to allow gas transfer. The system is sealed using Teflon® coated silicone seals and screw caps. Various designs developed (i–iv) with A, B, and C denoting the different chambers. The total volumes are as follows: i and ii (A: 10 mL, B: 50 mL), iii (A: 10mL, B: 50 mL, C: 50 mL), and iv (A: 50 mL, B: 50 mL). Glassware i was used when chamber A was run at room temperature, ii when chamber A was heated, iii when binding CO_2 in chamber C before releasing it in the closed system, and iv when conducting large scale reaction.

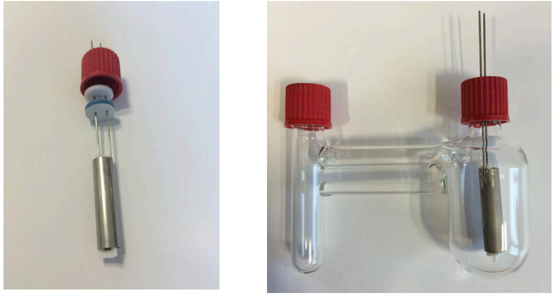

Supplementary Figure 15. The stainless steel electrodes were washed in aqua regia for 10 s followed by sonication for 5 min in water followed by acetone; the electrodes were left to dry. This efficient cleaning procedure allowed electrodes to be reused. Two Teflon® spacers were placed in each end of the two electrodes to keep them separated. By pinching a Teflon® coated silicone seal, the electrodes could be attached to a screw cap. Left picture shows the assembled electrode attached to a screw cap fitted with a Teflon® coated silicone seal. Right picture shows the glassware ii with the assembled electrode introduced and sealed with screw caps fitted with Teflon® coated silicone seals.

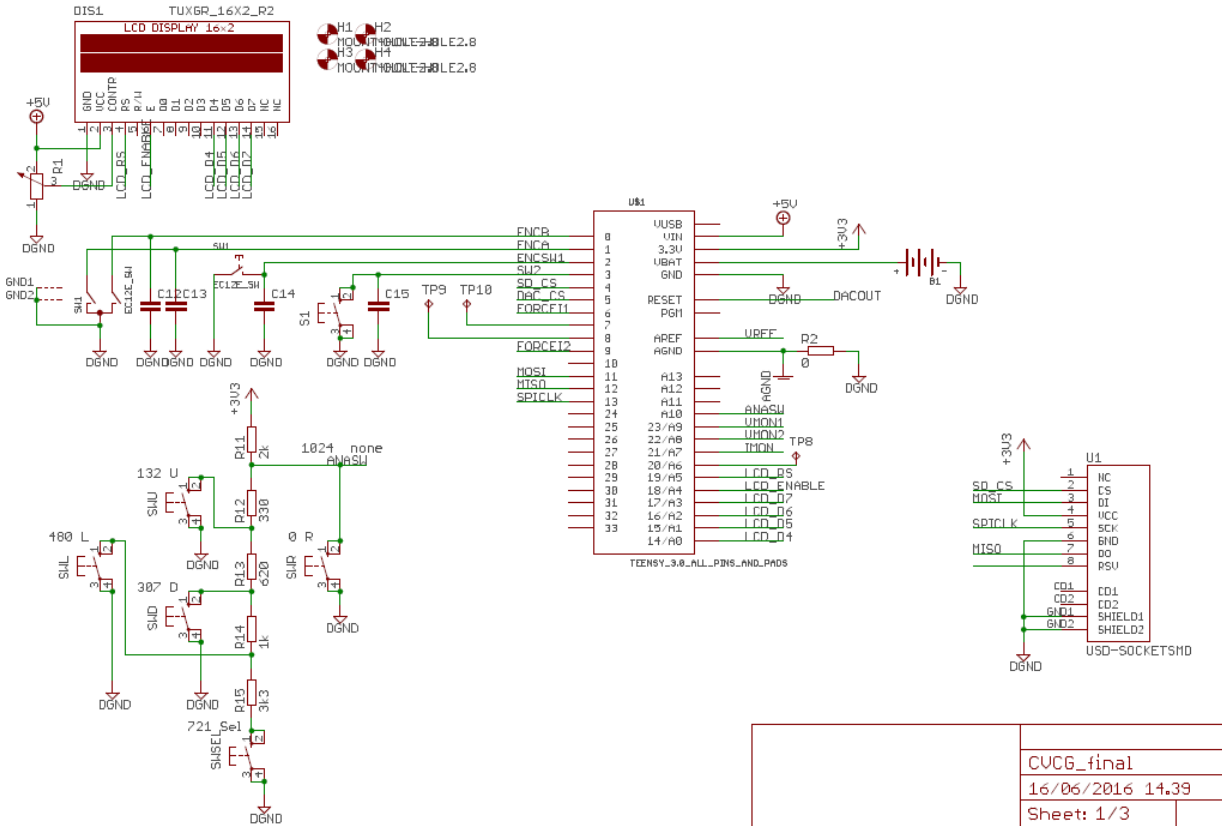

Supplementary Figure 16. Diagram showing the electronic components in the ElectroWare. The central unit is a small TEENSY 3.0 microprocessor that controls a potential and a current generator. The voltages and currents applied are measured independently of the generators and can be logged to a SD card. The ElectroWare is powered by either a 12 V battery or a 12 V power generator. Detailed information on software, calibration, and casings for the ElectroWare can be found on the URL: http://inano.au.dk/about/research-centers/cadiac/cadiac-electroware/

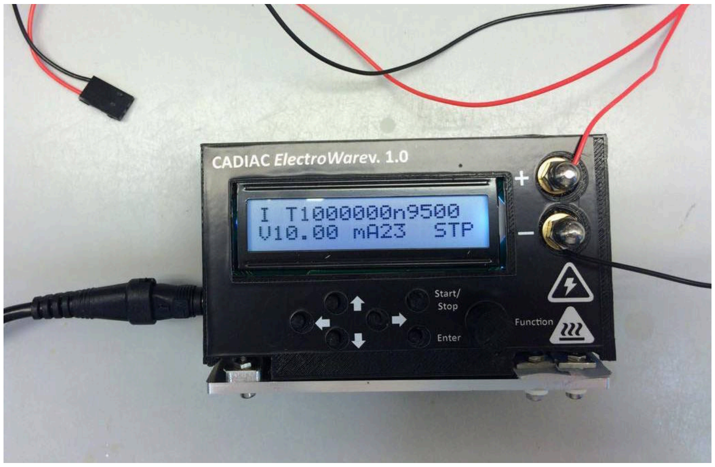

Supplementary Figure 17. The assembled ElectroWare.

```
[experiment_name                              = CADIAC], string i.e. >experiment name< max
length 16 char!
[experiment_date                              = dd mm year], string i.e. >12 12 2016< max
length 10 char!
[experiment_time                              = 00:00:00], string i.e. >23:59:00< max length 8
char!
[experiment_mode                              = 0], int i.e. XXX,  0 = current mode, 1 =
voltage mode
[experiment_voltageout_target                 = 5.00], float 0.000 to 10.00V (+-3.4028235 (E
+38) upto 6 digit)
[experiment_current_target                    = 23.00], float 0.0 - 200.0mA
[experiment_voltage_max_limit                 = 5.00], float 0.000 to 10.00V
[experiment_voltage_min_limit                 = 0.50], float 0.000 to 10.00V
[experiment_current_max_limit                 = 200.00], float 0.0 - 200.0mA
[experiment_current_min_limit                 = 0.01], float 0.0 - 200.0mA
[experiment_start_delay                       = 300000], unsigned long 0 - 1000000000mS (11
days)(4,294,967,295 upto 10 digit)
[experiment_running_time                      = 1000000000], unsigned long 0 - 1000000000mS
(11 days)(4,294,967,295 upto 10 digit)
[experiment_log_interval                      = 1000], unsigned long 100 - 100000mS
[experiment_mol_max_limit                     = xxxx], float 0.000 - 9999umol
[experiment_faradaic_efficiency               = 0.62], float 0.01 to 100%
[experiment_voltage_setpoint_current_mode = 10.00], float 0.000 to 10.00 normally 10.00V
[experiment_current_setpoint_voltage_mode = 200.00], float 0.000 - 200.0 normally 200.0mA
```

Supplementary Figure 18. Parameters used in Electroware E1 (controlled current experiments) which was set up on a computer using the micro SD-card. The parameters employed involve a constant current of 23 mA, voltage minimum of 0.50 V, voltage maximum of 5.00 V, start delay of 300000 ms, experiment running time of 1000000000 ms, and time log every 1000 ms. The maximum limit for the CO produced was set to 9999 µmol in the given case but, in general, varied depending on the exact reaction conditions.

```
[experiment_name                           = CADIAC], string i.e. >experiment name< max length 16 char!
[experiment_date                           = dd mm year], string i.e. >12 12 2016< max length 10 char!
[experiment_time                           = 00:00:00], string i.e. >23:59:00< max length 8 char!
[experiment_mode                           = 1], int i.e. XXX,  0 = current mode, 1 = voltage mode
[experiment_voltageout_target              = 3.60], float 0.000 to 10.00V (+-3.4028235 (E+38) upto 6 digit)
[experiment_current_target                 = 50.00], float 0.0 - 200.0mA
[experiment_voltage_max_limit              = 9.00], float 0.000 to 10.00V
[experiment_voltage_min_limit              = 0.50], float 0.000 to 10.00V
[experiment_current_max_limit              = 50.00], float 0.0 - 200.0mA
[experiment_current_min_limit              = 0.01], float 0.0 - 200.0mA
[experiment_start_delay                    = 300000], unsigned long 0 - 1000000000mS (11 days)(4,294,967,295 upto 10 digit)
[experiment_running_time                   = 1000000000], unsigned long 0 - 1000000000mS (11 days)(4,294,967,295 upto 10 digit)
[experiment_log_interval                   = 1000], unsigned long 100 - 100000mS
[experiment_mol_max_limit                  = 1500.0], float 0.000 - 9999umol
[experiment_faradaic_efficiency            = 0.62], float 0.01 to 100%
[experiment_voltage_setpoint_current_mode = 10.00], float 0.000 to 10.00 normally 10.00V
[experiment_current_setpoint_voltage_mode = 200.00], float 0.000 - 200.0 normally 200.0mA
```

Supplementary Figure 19. Parameters used in ElectroWare E2 (constant voltage experiments) which was set up on a computer using the micro SD-card. The parameters employed involve a constant voltage of 3.6 V, current minimum of 0.01 mA, current maximum of 50 mA, start delay of 300000 ms, experiment running time of 1000000000 ms, and time log every 1000 ms. The maximum limit for the CO produced was set to 1500 µmol.

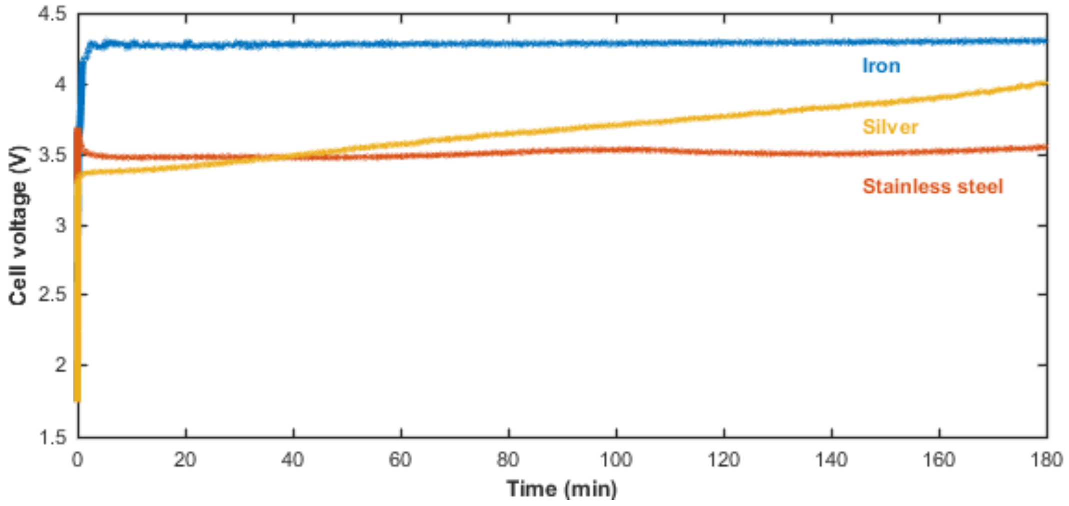

Supplementary Figure 20. Cell voltage recorded over time using three different cathode materials, i.e. stainless steel (orange), silver (yellow), and iron (blue), in the electrolysis (applied current = 23 mA) of a CO_2 saturated solution of 0.2 mM FeTPP and 0.8 M TFE in 0.1 M $TBABF_4$/DMF. For the stainless steel electrode during a three-hour constant-current electrolysis the variation in potential was within 50 mV after an initial stabilization period lasting <1 min. In comparison, for a silver electrode the potential changed with as much as 500 mV. With iron as electrode material a significant 700 mV increase in potential was required to keep the current constant.

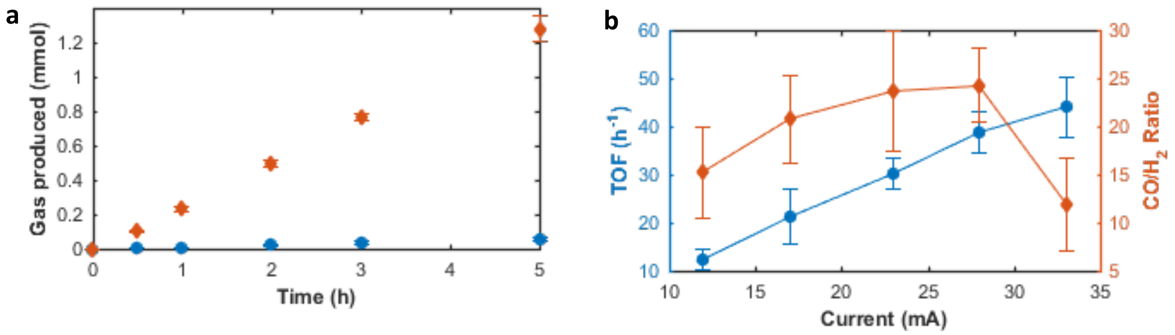

Supplementary Figure 21. a) Production of CO and H_2 over time using an applied current of 23 mA along with the b) turnover frequency (TOF) (blue) and ratio of CO_2/H_2 production (orange) as a function of current for the electrolysis on a CO_2 saturated solution of 0.2 mM FeTPP and 0.8 M TFE in 0.1 M $TBABF_4$/DMF. The faradaic efficiency (FE) increases slightly as the current is increased until 28 mA, where the highest CO/H_2 production ratio is attained. Error bars represent standard deviations for two independent experiments; the relative large uncertainty for the CO/H_2 ratio is due to the percentagewise large errors on the H_2 production due to the low production rate.

The FE is calculated from the best linear fit according to Supplementary Equation 1 below.

$$n_{\mathrm{CO}} = \frac{Q}{n_{\mathrm{e}}F}\mathrm{FE} \qquad \text{(Supplementary Equation 1)}$$

In this expression n_{CO} denotes the number of moles of CO produced, Q is the charge, F is Faraday´s constant, and n_{e} is the number of electrons involved in the electroreduction of one CO_2 molecule to CO (i.e. n_{e} = 2). In the given case the slope = 3.19×10^{-3} mmol C^{-1} (Supplementary Figure 22) which corresponds to FE = 62%.

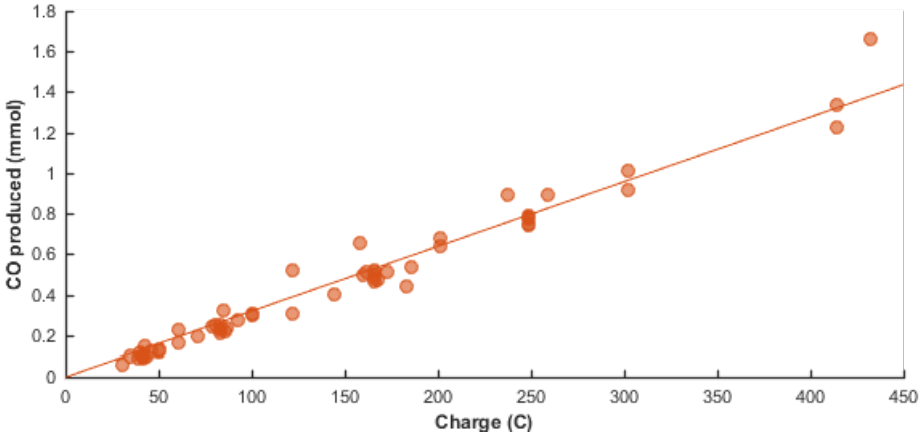

Supplementary Figure 22. Production of CO as a function of the charge consumed in electrolyses using applied currents ranging from 17–28 mA on CO_2 saturated solutions of 0.2 mM FeTPP and 0.8 M TFE in 0.1 M $TBABF_4$/DMF.

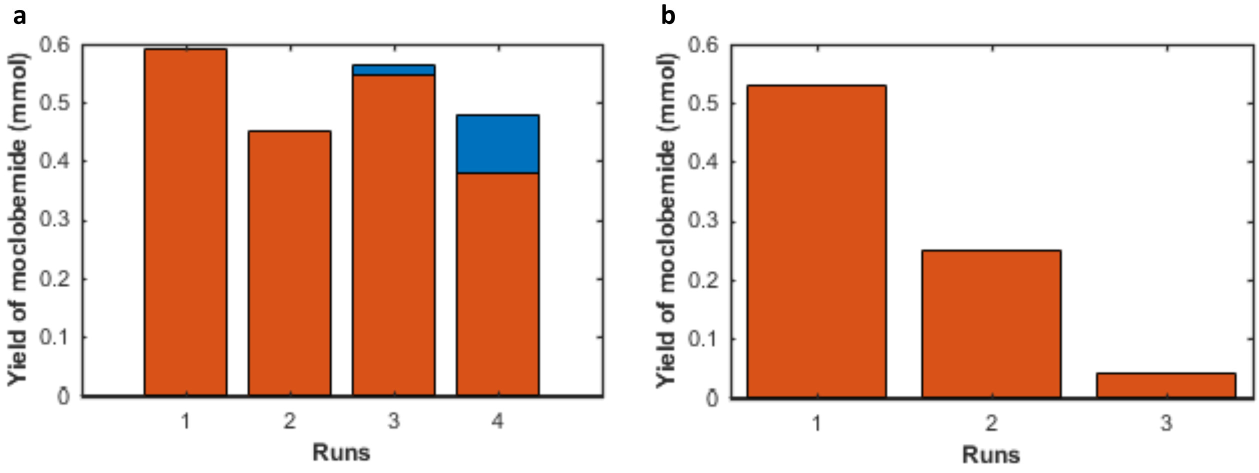

Supplementary Figure 23. Test of the reusability of the electrochemical set-up by repeating multiple times the reaction sequence and analysis of products in the carbonylation reaction, when a) reusing

electrodes and b) mounting electrodes outside the glovebox. The reaction mixture in the chemical reaction chamber consisted of 3.0 mmol 1-chloro-4-iodobenzene, 6.0 mmol amine **3**, 5 mol% Xantphos Pd G4, and 6.0 mmol DABCO; the electrochemical chamber contained CO_2 saturated 0.1 M $TBABF_4$/DMF with 5 mg FeTPP and 2 mL TFE added. The ElectroWare was set to produce 0.5 mmol of CO and the yield of moclobemide (orange column) generated was determined by HPLC analysis of an aliquot from the chemical reaction chamber. Some minor reduction of 1-chloro-4-iodobenzene to chlorobenzene (blue column) was observed for runs 3 and 4 in a).

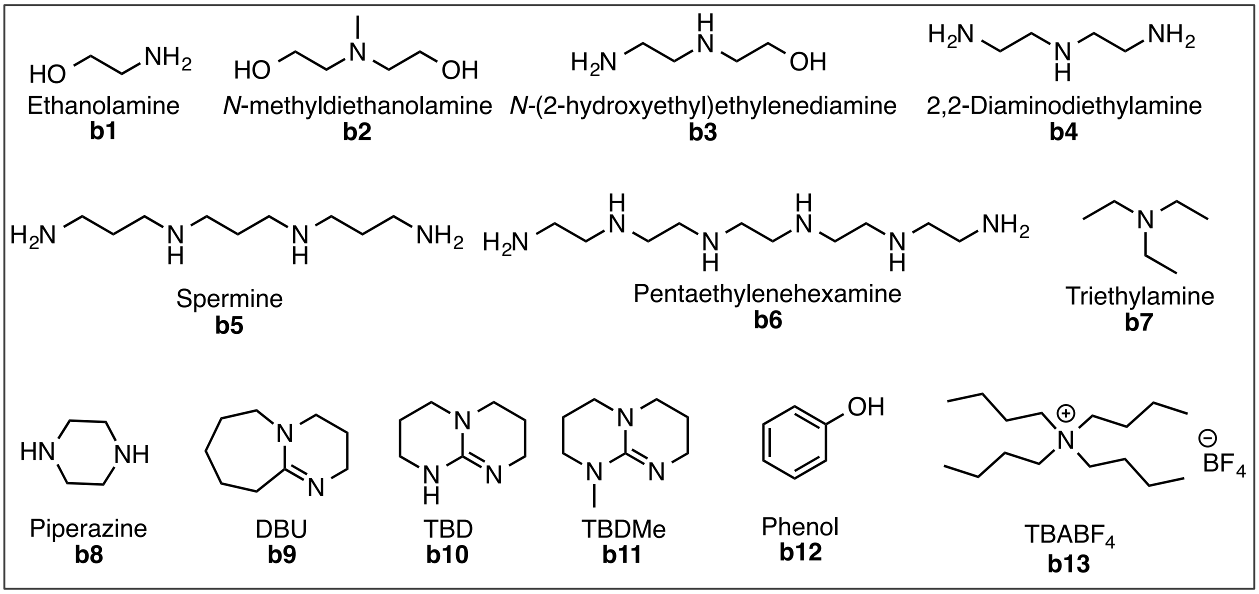

Supplementary Figure 24. Structure of the different CO_2 binders that were tested.

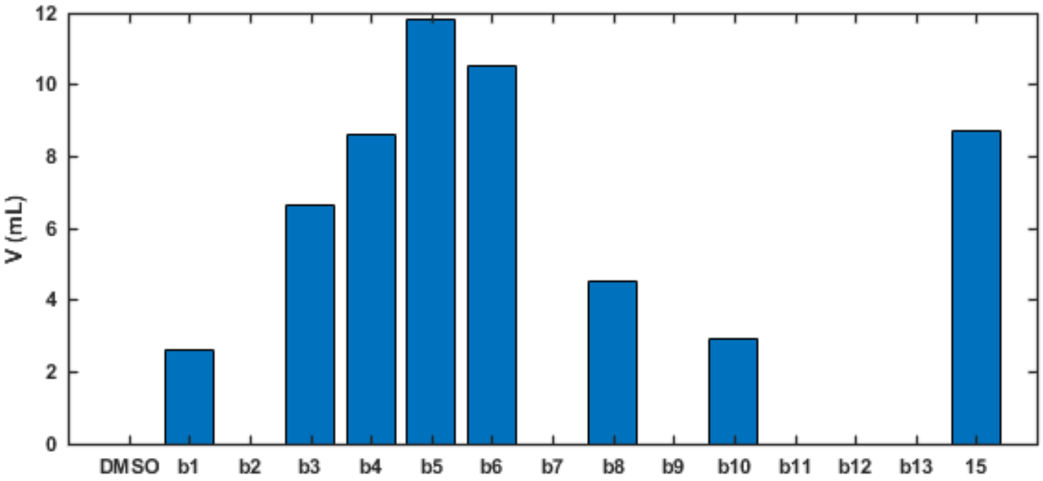

Supplementary Figure 25. CO_2 released from the different CO_2 binders in DMSO. In a flame-dried 10 mL vial equipped with a magnetic stirring bar the given binder (0.5 mmol) was dissolved in DMSO (3 mL). The vial was sealed with a screw cap fitted with a Teflon® coated silicone seal. The solution of each compound was bubbled with Ar for 10 min followed by CO_2 for 10 min and, finally, Ar for 1 min to remove excess CO_2 in solution. The solution was then heated to 130 °C and the CO_2 released determined using syringes (blue). The theoretical volume of 0.5 mmol CO_2 is 11.1 mL at 1 bar. The CO_2 binders **b3–b6** and **15** demonstrated the highest efficiency. Compound **15** was chosen as the CO_2 binder for further study due to problems with reproducibility and undesired precipitation of simple polyamines in the case of **b3–b6**.

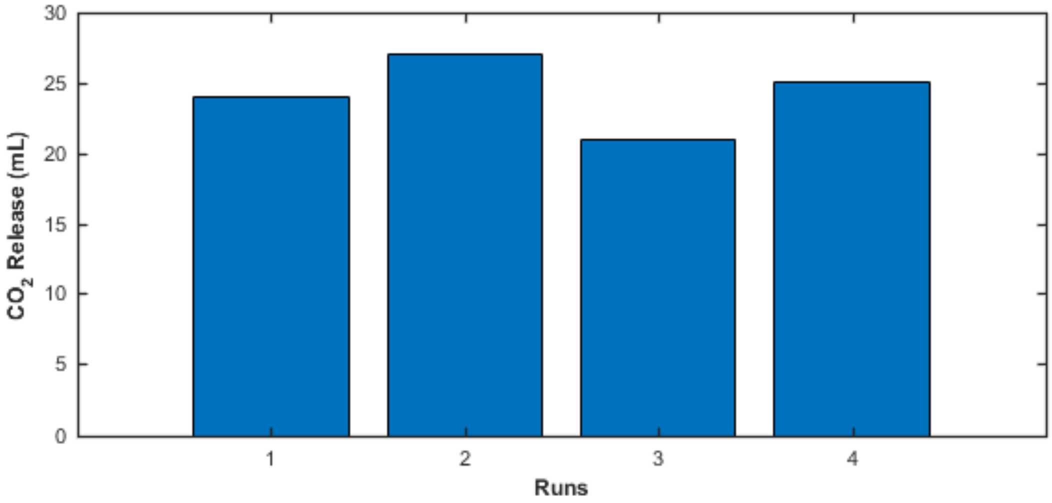

Supplementary Figure 26. Reusability of **15** in a DMSO solution. In a flame-dried 10 mL vial **15** (0.5 mmol) was dissolved in DMSO (3 mL). The vial was sealed using a screw cap fitted with a Teflon® coated silicone seal. The solution was bubbled with Ar for 10 min followed by CO_2 for 10 min and, finally, Ar for 1 min to remove excess CO_2 dissolved in DMSO. The solution was heated to 130 °C for 20 min. The

amount of CO_2 released was determined using syringes (blue). The solution was reused four times giving similar results after every run.

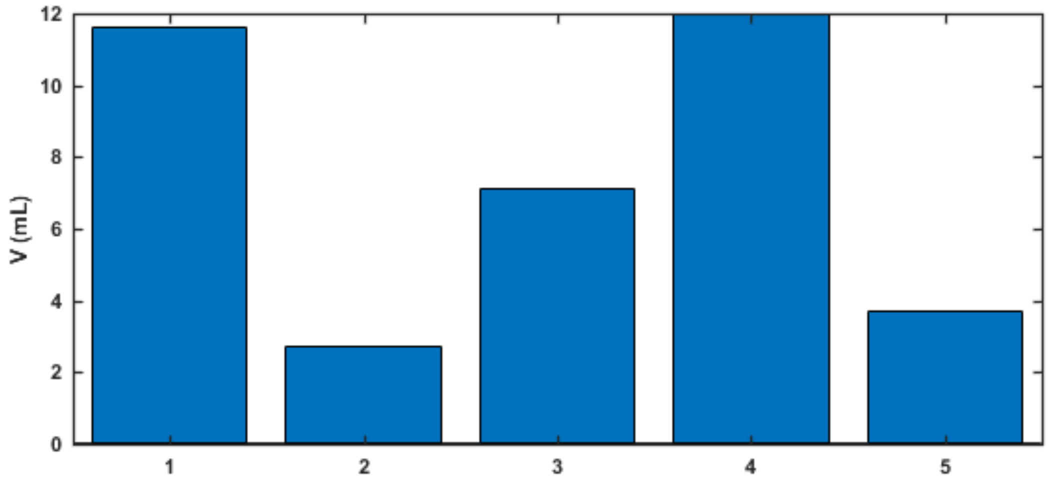

Supplementary Figure 27. Addition of CO_2 using different sources. In a flame-dried 10 mL vial **15** (0.5 mmol) was dissolved in DMSO (3 mL). The vial was sealed using a screw cap fitted with a Teflon® fitted silicone seal. In (1) pure CO_2 (20 mL) was injected using a syringe. In (2) and (3) air was bubbled through molecular sieves to avoid water in the solution for 1 day (2) and 2 days (3). In (4) air was bubbled through a fine filter into the solution for 1 day, and in (5) a balloon with exhalation air was placed on top of the solution. All solutions were bubbled with argon for 1 min to remove excess CO_2 dissolved in the solution. The solutions were heated to 130 °C for 20 min and CO_2 release determined using syringes. The releases of CO_2 from the solution when using pure CO_2 compared to air through a fine filter are comparable, which indicate a good selectivity for binding CO_2.

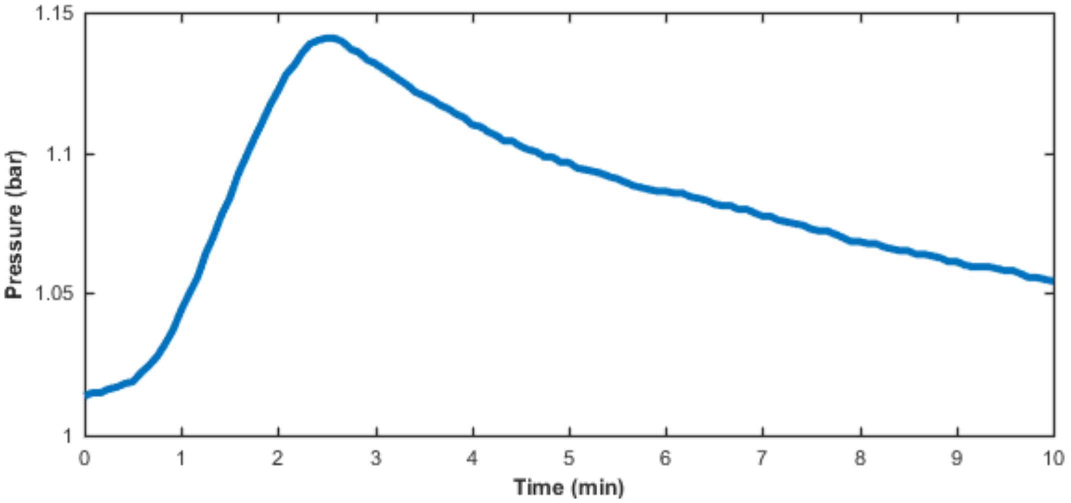

Supplementary Figure 28. Pressure release curve for the release of CO_2 from **15** in one chamber and the solubility of CO_2 in DMF contained in the other chamber. In a flame-dried glassware i, **15** (0.5 mmol) was dissolved in DMSO (3 mL) in chamber B. In chamber A DMF (3 mL) was added. Chamber B was sealed

with a screw cap fitted with a Teflon® coated silicone seal. A Keller manometer was used to seal chamber B. Chamber B was heated to 130 °C until the pressure was constant in the system. As the released CO_2 was dissolved in DMSO in chamber B, the pressure decreased.

Supplementary Table 1. Charge consumption and rates of CO and H_2 production from 1 h electrolysis of CO_2 in three control experiments performed in the absence of TFE or FeTPP. No CO was detectable, while H_2 was produced in faradaic yields of 25–44%.[a]

Entry	Charge consumption (C)	CO (mmol h^{-1})	H_2 (mmol h^{-1})
1 [b]	10	0.00	0.01
2 [c]	88	0.00	0.20
3 [d]	83	0.00	0.18

[a] Electrolysis solution consists of 35 mL CO_2 saturated 0.1 M $TBABF_4$/DMF . [b] Applied voltage = 3.8 V with added FeTPP (0.2 mM). [c] Applied voltage = 3.8 V with added TFE (0.8 M). [d] Applied current = 23 mA with added TFE (0.8 M).

Supplementary Table 2. Screening of conditions when using atmospheric air as CO_2 source. The General Procedure 1 with glassware iii and the setup for the ElectroWare E2 (Supplementary Figure 19) were followed.

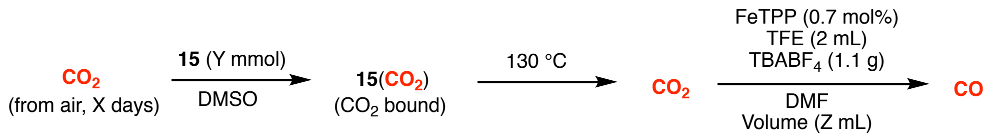

Entry	Air (X days)	**15** (Y mmol)	Size of Chamber B (Z mL)	CO amount set on Electroware (mmol)	Estimated yield of carbonylation product, moclobemide (Isolated yield)
1	10	5.0	50	2.5	>99%[a] (99%)
2	2	5.0	50	2.5	22%[a]
3	4	5.0	50	2.5	88%[a]
4	6	5.0	50	2.5	>99%[a] (99%)
5	6	5.0	50	0.75	60% [a]
6	6	5.0	20	0.75	0%[a]
7	6	2.0	50	2.5	69%[a]
8	6	2.0	50	1.5	17%[a]
9	6	5.0	50	1.5	>99%[a]

[a] Yields estimated from the ratio of remaining starting material and product from ^1H NMR analysis.

Supplementary Methods

Materials

Iron(III) *meso*-tetraphenylporphine chloride (FeTPP) was purchased from ABCR (AB120710) and Sigma Aldrich (259071). 2,2,2-Trifluoroethanol (TFE) was obtained from Sigma Aldrich (91683). Tetrabutylammonium tetrafluoroborate ($TBABF_4$) was prepared by mixing a 1:1 molar ratio of sodium tetrafluoroborate purchased from Sigma Aldrich (202215) and tetrabutylammonium hydrogensulfate from Fluka (86853). The precipitate was recrystallized in ethyl acetate and pentane, and dried under vacuum at 80 °C.[1] Glassy carbon (GC) rods (Sigradur G) were supplied by HTW Hochtemperatur-Werkstoffe Gmbh. The Ag/AgCl reference electrode with a filling electrolyte of 3.4 M KCl was purchased from ElectroCell. Diamond paste was supplied from Struers. Stainless steel 316 was obtained as cylinders from Sanistål and modified in our own workshop to obtain the desired electrodes. Silver (99.95% purity) was purchased as foil from GoodFellow.

Solvents were dried according to standard procedures and flash column chromatography was carried out on silica gel 60 (230–400 mesh). The chemical shifts are reported in ppm relative to solvent residual peak. The ^{1}H NMR spectra were recorded at 400 MHz, ^{13}C NMR spectra at 100 MHz, ^{19}F NMR spectra at 367 MHz on a Bruker 400 spectrometer. NMR spectra are reported as follows: s = singlet, d = doublet, t = triplet, q = quartet, quin = quintuplet, sext = sextet, sep = septet, m = multiplet, br = broad, dd = double doublet, dt = double triplet, ddd = double double doublet; coupling constant(s) in Hz. HRMS spectra were recorded on a LC TOF (ES) apparatus. HPLC analysis was performed on a reverse phase Jupiter 5u C18 300 A 250 × 10.00 mm 5 micron column with a linear gradient 5–90% CH_3CN in milliQ water (1% TFA) employed over 25 min at 215 nm. All experiments were performed in the glassware described in Supplementary Figure 14.

Glassware under pressure - Warning!

- Glass equipment should always be examined for damages to its surface, which may weaken its strength.
- One must abide to all laboratory safety procedures and always work behind a shield when working with glass equipment under pressure.
- This glassware is pressure tested to 224 psi, but should under no circumstances be operated above 60 psi (5 bar).

General Procedure for Cyclic Voltammetry

All experiments were performed in a three-necked sealed flask using a three-electrode setup. The working electrode was either a glassy carbon (GC) disk electrode (Ø = 0.1 cm) or a stainless steel disk electrode (Ø = 0.3 cm). Before performing experiments the working electrode was polished using diamond paste of various grain sizes, depending on the exact electrode material, i.e. stainless steel (grain size: 3 µm) and GC (grain size: 9, 3, 1, and 0.25 µm), followed by sonication in acetone for 10 min. A stainless steel cylinder (length = 4 cm, outer diameter = 4 mm, and inner diameter = 2 mm) served as counter electrode and Ag/AgCl was used as reference electrode. The stainless steel working electrode was pre-activated by running 50 continuous cyclic voltammograms between –1 and –2 V vs Ag/AgCl in 0.1 M $TBABF_4$/DMF prior to the actual experiment. All voltammetric recordings were performed with a CHI 601D potentiostat using a sweep rate (ν) of 100 mV s^{-1}.

General Procedure for Electrosynthesis

All optimization experiments were performed in a glassware i two-chamber (see Supplementary Figure 14) with the solution placed in chamber B (volume = 35 mL) and using a two-electrode setup. The stainless steel working electrode (cathode) was cylindrically shaped (length = 4 cm, outer diameter = 10 mm, and inner diameter = 9 mm; surface area = 23.9 cm^2). The counter electrode (anode) was a stainless steel cylinder (length = 4 cm, outer diameter = 4 mm, and inner diameter = 2 mm; surface area = 7.5 cm^2). A small Teflon® spacer separated the two electrodes (see Figure 2a iv). Galvanostatic electrolysis was performed using an Autolab PGSTAT302 or the homebuilt ElectroWare *(vide supra*). Constant voltage electrolysis utilized a CHI 601D or the ElectroWare.

General Procedure for Gas Detection

Gas analysis was performed on an Agilent 7890B Gas Chromatograph equipped with a tandem column and further connected to a TCD detector for quantification of the gases produced. The tandem column–one part being based on 5 Å molecular sieves and the other on PoraBOND Q–serves the role of separating the permanent gases and CO_2. The detector temperature was kept at 200 °C and the oven at 45 °C. The carrier gas was argon flowing at 14 mL min^{-1}. The quantification of CO and H_2 was based on a calibration curve obtained by injecting known volumes of a calibration gas consisting of 20% CO_2, 5% CO, 5% methane, 2% H_2, and 1% ethane mixed in argon. Gas samples were injected by a 250 µL Hamilton syringe. The total volume of the gas phase was determined as the sum of that of the gas phase above the liquid (= 15 mL) in chamber B plus the volume of chamber A (10 mL) in the glassware i design and the expansion of the piston in a 20 mL syringe when pinched into chamber A after the experiment.

General Procedure 1 (Figure 4)

In a flame-dried two-chamber charged with stirring bars were added Xantphos Pd G4 (24 mg, 5 mol%), aryl bromide (0.5 mmol), dioxane (3 mL), Et_3N (139 μL, 1.0 mmol), and amine (1.5 equiv.) were added to chamber A (see specific reaction conditions in the Characterization of Products section). FeTPP (6 mg), $TBABF_4$ (1.10 g), DMF (35 mL), and TFE (2 mL) were introduced to chamber B. Electrodes were mounted and the glassware was sealed with screw caps fitted with Teflon® coated silicone seals. The solution in chamber B was bubbled through with CO_2 for 10–15 min (outlet located in chamber A). The ElectroWare was set up using the galvanostatic configuration E1, electrodes were connected, and electrolysis was commenced while stirring the solution in both chambers. Chamber B was at room temperature while chamber A was placed in a preheated heat block. The reactions were stirred for 18 h. All yields are average of two runs.

General Procedure 2 (Figure 4)

In a flame-dried two-chamber charged with stirring bars FeTPP (6 mg), $TBABF_4$ (1.10 g), DMF (20 mL), and TFE (2 mL) were added to chamber B. Electrodes were mounted and sealed off with a screw cap fitted with a Teflon® coated silicone seal. The glassware was transferred to an argon filled glovebox. Palladium catalyst, ligand, aryl bromide, base, solvent and amine were added to chamber A (see specific reaction conditions in the Characterization of Product section), which was then sealed with a screw cap fitted with a Teflon® coated silicone seal before being removed from the glovebox. Additional DMF (15 mL) was added to chamber B and the solution bubbled through with CO_2 for 10–15 min to achieve saturation (outlet located in chamber A). The ElectroWare was set up using the galvanostatic configuration E1, electrodes were connected, and electrolysis was commenced while stirring the solution in both chambers. Chamber B was kept at room temperature while chamber A was placed in a preheated heat block. The reactions were stirred for 18 h. All yields are average of two runs.

General Procedure 3 (Figure 3d)

In a flame-dried glassware i two-chamber charged with stirring bars Xantphos Pd G4 (289 mg, 0.15 mmol), 1-chloro-4-iodobenzene (715 mg, 3 mmol), DABCO (673 mg, 6 mmol), THF (18 mL), and 2-morpholinoethylamine (880 μL, 6 mmol) were added to chamber A. FeTPP (6 mg), $TBABF_4$ (1.10 g), DMF (35 mL), and TFE (2 mL) were introduced to chamber B. Electrodes were mounted in chamber B and the glassware sealed with screw caps fitted with Teflon® coated silicone seals. The solution in chamber B was bubbled through with CO_2 for 10–15 min to achieve saturation (outlet located in chamber A). The ElectroWare was set up using the galvanostatic configuration E1 with the parameter pertaining to the amount of produced CO fixed at a given value in the range 250–3000 μmol, electrodes were connected, and electrolysis was commenced while stirring the solution in both chambers at room temperature. After end CO production–controlled by the ElectroWare–the reaction was stirred at room temperature for additional 12 h. Acetanilide (1 equiv.) was added as internal standard before the reaction mixture was analysed and yield estimated by HPLC analysis of a 30 μL aliquot diluted in acetonitrile. Retention time of 1-chloro-4-iodobenzene, 4-chloro-N-(2-morpholinoethyl)benzamide

(moclobemide) (**1**), chlorobenzene, and acetanilide (internal standard) was 15.4, 7.9, 13.3, and 7.7 min, respectively. All yields are average of two runs.

General Procedure 4 (Figure 3e)

In a flame-dried glassware iv two-chamber charged with stirring bars Xantphos Pd G4 (144 mg, 0.15 mmol), 1-chloro-4-iodobenzene (715 mg, 3 mmol), DABCO (673 mg, 6 mmol), acetanilide as internal standard (135 mg, 1 mmol), THF (18 mL), and 2-morpholinoethylamine (880 μL, 6 mmol) were introduced to chamber A. FeTPP (6 mg), $TBABF_4$ (1.1 g), DMF (35 mL), and TFE (2 mL) were introduced to chamber B. Electrodes were mounted in chamber B and the glassware sealed with screw caps fitted with Teflon® coated silicone seals. The solution in chamber B was bubbled through with CO_2 for 10–15 min to achieve saturation (outlet located in chamber A). The ElectroWare was set up using the galvanostatic configuration E1 with the parameter pertaining to the amount of produced CO fixed at 500 μmol, electrodes were connected, and electrolysis was commenced while stirring the solution in both chambers at room temperature. After end CO production–controlled by the ElectroWare–the reaction was stirred at room temperature for additional 12 h. After every run the glassware was transferred to an argon filled glovebox and a 30 μL sample from chamber A was removed for analysis by HPLC. The electrodes in chamber B were changed and the glassware sealed with new screw caps fitted with new Teflon® coated silicone seals before it was removed from the glovebox. Chamber B was saturated with CO_2, the ElectroWare was attached to the electrodes, and electrolysis commenced under stirring. The reaction was run again at room temperature for 12 h. This last part was repeated after every run–in a total of 4 times.

General Procedure 5 (Figure 5)

In a 50 mL vial N^2,N^6-bis(2-((2-aminoethyl)amino)ethyl)pyridine-2,6-dicarboxamide (**15**) (1.69 g, 5.0 mmol) was dissolved in DMSO (30 mL). The solution was bubbled with air (through molecular sieves) for 6 days. FeTTP (6 mg) and $TBABF_4$ (1.10 g) were introduced to chamber B and Xantphos Pd G4 (24 mg, 0.025 mmol), DABCO (112 mg, 1.0 mmol), and 1-chloro-4-iodobenzene (119 mg, 0.5 mmol) to chamber C in glassware iii. The DMSO solution of **15** was transferred to chamber A. To chamber C was added THF (3 mL) and 2-morpholinoethylamine (131 μL, 1.0 mmol). To chamber B was added DMF (35 mL) and TFE (2 mL). The electrodes were mounted in chamber B and the glassware sealed using screw caps fitted with Teflon® coated silicone seals. The ElectroWare was set up using configuration E2 to control the voltage and electrodes were connected. Chamber A was heated to 130 °C for 20 min before the electrolysis was commenced. The solutions in chambers B and C were stirred at room temperature for 20 h. Column chromatography (2% MeOH in CH_2Cl_2) yielded moclobemide as a white solid (133 mg, 99%).

Procedure for Conducting Large Scale Reaction

In a flame-dried glassware iv charged with stirring bars was added Xantphos Pd G4 (0.578 g, 0.6 mmol), 1-chloro-4-iodobenzene (1.43 g, 6 mmol), DABCO (1.35 g, 12 mmol), THF (36 mL) and 2-morpholinoethylamine (1.76 mL, 12 mmol) to chamber A and FeTPP (6 mg), $TBABF_4$ (1.1 g), DMF (35 mL) and TFE (2 mL) to chamber B. The electrodes were mounted in chamber B and the glassware sealed with screw caps fitted with Teflon® coated silicone seals. The solution in chamber B was bubbled through with CO_2 for 10–15 min to achieve saturation (outlet located in chamber A). The ElectroWare was set up using the galvanostatic configuration E1 with the parameter pertaining to the amount of produced CO fixed at 6000 μmol, electrodes were connected, and electrolysis was commenced while stirring the solution in both chambers. After end CO production–controlled by the ElectroWare–the reaction was stirred at room temperature for additional 12 h. Column chromatography (2% MeOH in CH_2Cl_2) followed by recrystallization in heptane and CH_2Cl_2 afforded moclobemide as a white solid (1.27 g, 79%).

Characterization of Products

4-Chloro-*N*-(2-morpholinoethyl)benzamide (Moclobemide) (1)

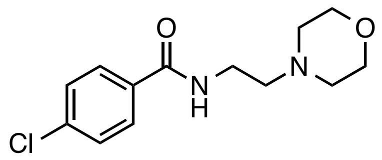

1 was prepared using General Procedure 1 in glassware i. In chamber A was added 1-chloro-4-iodobenzene (112 mg, 0.5 mmol), Xantphos Pd G4 (24 mg, 0.025 mmol), DABCO (119 mg, 1.0 mmol), THF (3 mL), and 2-morpholinoethylamine (113 μL, 1.0 mmol). The ElectroWare was set to produce 750 μmol CO. The reactions were stirred at rt for 5 h. Column chromatography (2% MeOH in CH_2Cl_2) afforded a grey solid (130 mg, 97%). ^1H NMR (400 MHz, $CDCl_3$) δ 7.70 (d, *J* = 8.5 Hz, 2H), 7.41 (d, *J* = 8.5 Hz, 2H), 6.74 (bs, 1H), 3.72 (t, *J* = 4.5 Hz, 4H), 3.53 (q, *J* = 5.6 Hz, 2H), 2.59 (t, *J* = 6.0 Hz, 2H), 2.49 (t, *J* = 4.4 Hz, 4H). ^{13}C NMR (100 MHz, $CDCl_3$) δ 166.4, 137.7, 133.1, 128.9 (2C), 128.5 (2C), 67.1 (2C), 56.9, 53.5 (2C), 36.2. HRMS $C_{13}H_{18}ClN_2O_2$ [M+H$^+$]: calculated 269.1051, found 269.1056.
The data were in accordance to literature.[2]

4-(3-(4-(Cyclopropanecarbonyl)piperazine-1-carbonyl)-4-fluorobenzyl)phthalazin-1(2*H*)-one (Olaparib) (4)

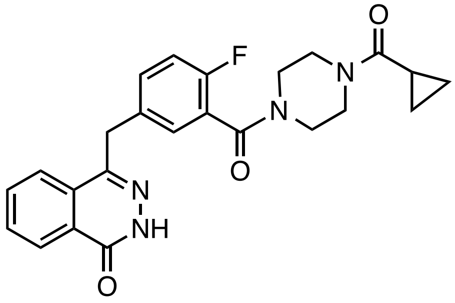

4 was prepared using General Procedure 1 in glassware ii. In chamber A was added 4-(3-bromo-4-fluorobenzyl)phthalazin-1(2*H*)-one (166 mg, 0.5 mmol), cyclopropyl(piperazin-1-yl)methanone (154 mg, 1.0 mmol), Xantphos Pd G4 (24 mg, 0.025 mmol), dioxane (3.0 mL), and DIPEA (125 μL, 1.4 mmol). The ElectroWare was set to produce 750 μmol CO. Chamber A was stirred at 100 °C. Column chromatography (1% MeOH in EtOAc) afforded a light yellow solid (187 mg, 86 %). ^1H NMR

(400 MHz, $CDCl_3$) δ 11.5 (bs, 1H), 8.51-8.42 (m, 1H), 7.82–7.65 (m, 3H), 7.41–7.28 (m, 2H), 7.02 (t, J = 8.8 Hz, 1H), 4.29 (s, 2H), 3.93–3.68 (m, 4H), 3.50-3.43 (m, 2H), 3.43–3.32 (m, 2H), 1.83-1.55 (m, 1H), 1.05-0.93 (m, 2H), 0.88-0.65 (m, 2H). ^{13}C NMR (100 MHz, $CDCl_3$) δ 172.4, 165.3, 161.0, 157.1 (d, J = 247.5 Hz), 145.6, 134.5 (d, J = 3.4 Hz), 133.7, 131.8 (d, J = 8.0 Hz), 131.7, 129.6, 129.3 (m), 128.4, 127.2, 125.1, 123.8 (d, J = 17.6 Hz), 116.3 (d, J = 22.1 Hz), 47.2, 46.9, 45.8, 45.2, 42.4, 37.8, 11.1, 7.8. ^{19}F NMR (367 MHz, $CDCl_3$) δ –117.7. HRMS $C_{24}H_{24}FN_4O_3$ [M+H$^+$]: calculated 435.1827, found 435.1830.
The data were in accordance to literature.[3]

(*Z*)-*N*-(2-(Diethylamino)ethyl)-5-((5-fluoro-2-oxoindolin-3-ylidene)methyl)-2,4-dimethyl-1*H*-pyrrole-3-carboxamide (Sunitinib) (5)

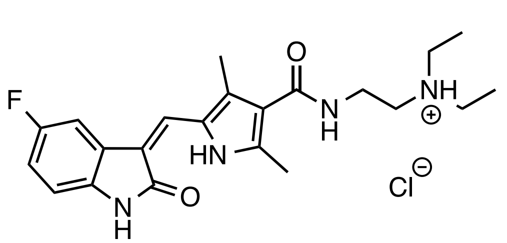

5 was prepared using General Procedure 2 in glassware ii. In chamber A was added (*Z*)-5-fluoro-3-((4-iodo-3,5-dimethyl-1*H*-pyrrol-2-yl)methylene)indolin-2-one (115 mg, 0.3 mmol), $Pd(dba)_2$ (13 mg, 0.0225 mmol), Xantphos (13 mg, 0.0225 mmol), dioxane (3 mL), Et_3N (126 μL, 0.9 mmol), and *N,N*-diethylethylene-1,2-diamine (127 μL, 0.9 mmol). The ElectroWare was set to produce 450 µmol CO. Chamber A was stirred at 80 °C. Column chromatography (0–10% MeOH in $CHCl_3$) followed by precipitation in absolute EtOH using HCl in EtOH (1 mL, 1M). The solution was refluxed before concentrated to saturation and cooled to rt. The mixture was left in the freezer at –21 °C before the product could be filtered off as an orange solid (83 mg, 66%). ^1H NMR (400 MHz, DMSO-d_6) δ 13.74 (s, 1H), 10.93 (s, 1H), 10.11 (bs, 1H), 7.97–7.89 (m, 1H), 7.77 (dd, J = 9.4, 2.3 Hz, 1H), 7.72 (s, 1H), 6.93 (td, J = 9.2, 2.4 Hz, 1H), 6.88-6.81 (m, 1H), 3.66–3.55 (m, 2H), 3.28–3.11 (m, 6H), 2.48 (s, 3H), 2.46 (s, 3H), 1.25 (t, J = 7.2 Hz, 6H). ^{13}C NMR (100 MHz, DMSO-d_6) δ 169.5, 165.3, 158.2 (d, J = 234.3 Hz), 137.0, 134.6 (d, J = 1.0 Hz), 130.3, 127.1 (d, J = 9.5), 125.8, 124.8, 119.6, 115.0 (d, J = 3.1 Hz), 112.5 (d, J = 24.2 Hz), 110.1 (d, J = 8.6 Hz), 106.0 (d, J = 25.7), 50.1, 46.9 (2C), 34.0, 13.6, 10.8, 8.5 (2C). ^{19}F NMR (367 MHz, DMSO-d_6) δ –122.5. HRMS $C_{22}H_{28}FN_4O_2$ [M+H$^+$]: calculated 399.2191, found 399.2195.
The data were in accordance to literature.[4]

***N*-(4-(2-(Dimethylamino)ethoxy)benzyl)-3,4-dimethoxybenzamide (Itopride) (6)**

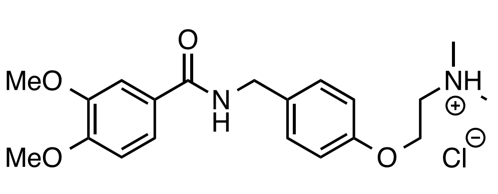

6 was prepared using General Procedure 1 in glassware ii. In chamber A was added 4-bromoveratrole (109 mg, 0.5 mmol), Xantphos Pd G4 (24 mg, 0.025 mmol), dioxane (3 mL), Et_3N (139 μL, 1 mmol) and 2-(4-(aminomethyl)phenoxy)-*N,N*-dimethylethan-1-amine (194 mg, 1 mmol). The ElectroWare was set to produce 750 µmol CO. Chamber A was stirred at 80 °C. Column chromatography (CH_2Cl_2/EtOAc/MeOH, 5:1:1) followed by precipitation in absolute EtOH using HCl in EtOH (1 mL, 1 M). The solution was refluxed before concentrated to saturation and cooled to rt. The mixture was left in the freezer at –21 °C before the product was filtered off as a colourless solid (124 mg, 63%). ^1H NMR (400 MHz, DMSO-d_6) δ 11.03 (s, 1H), 9.03 (t, J = 5.9 Hz, 1H), 7.58–7.51 (m, 2H), 7.28 (d, J = 8.5 Hz, 2H),

7.01 (d, J = 8.2 Hz, 1H), 6.95 (d, J = 8.5 Hz, 2H), 4.40 (d, J = 5.8 Hz, 2H), 4.35 (t, J = 5.1 Hz, 2H), 3.80 (s, 3H), 3.80 (s, 3H), 3.51–3.44 (m, 2H), 2.80 (d, J = 2.9 Hz, 6H). ^{13}C NMR (100 MHz, (DMSO-d_6) δ 165.5, 156.4, 151.2, 148.2, 132.9, 128.7 (2C), 126.6, 120.5, 114.5 (2C), 110.8, 110.7, 62.4, 55.6 (2C), 55.1, 42.6 (2C), 42.0. HRMS $C_{20}H_{27}N_2O_4$ [M+H$^+$]: calculated 359.1965, found 359.1964.

(*S*)-*N*-((1-Ethylpyrrolidin-2-yl)methyl)thiophene-2-carboxamide (7)

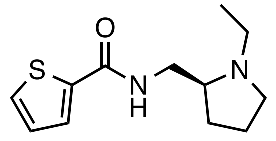

7 was prepared using General Procedure 1 in glassware ii. In chamber A was added 2-bromothiophene (82 mg, 0.5mmol), Xantphos Pd G4 (24 mg, 0.025 mmol), (*S*)-(1-ethylpyrrolidin-2-yl)methanamine (77 mg, 0.6 mmol), dioxane (3 mL) and Et_3N (139 μL, 1.4 mmol). The ElectroWare was set to produce 750 μmol CO. Chamber A was stirred at 80 °C. Column chromatography (0–1% Et_3N in CH_2Cl_2) afforded a light yellow solid (100 mg, 84%). ^{1}H NMR (400 MHz, $CDCl_3$) δ 7.49 (d, J = 3.6 Hz, 1H), 7.45 (d, J = 5.0 Hz, 1H), 7.07 (dd, J = 5.0, 3.7 Hz, 1H), 6.70 (bs, 1H), 3.65 (ddd, J = 13.6, 7.3, 2.8 Hz, 1H) 3.35–3-25 (m, 1H), 3.24–3.16 (m, 1H), 2.83 (dq, J = 11.9, 7.4 Hz, 1H), 2.68 (bs, 1H), 2.34–2.14 (m, 2H), 1.97–1.85 (m, 1H), 1.73 (q, J = 8.2 Hz, 2H), 1.68–1.56 (m, 1H), 1.13 (t, J = 7.2 Hz, 3H). ^{13}C NMR (100 MHz, $CDCl_3$) δ 162.3, 139.5, 129.7, 127.9, 127.7, 62.3, 53.8, 48.3, 41.0, 28.4, 23.2, 14.3. HRMS $C_{12}H_{19}N_2OS$ [M+H$^+$]: calculated 239.1213, found 239.1219.

(4-Isopropylpiperazin-1-yl)(4-((tetrahydro-2*H*-pyran-4-yl)oxy)phenyl)methanone (H3-receptor antagonist) (8)

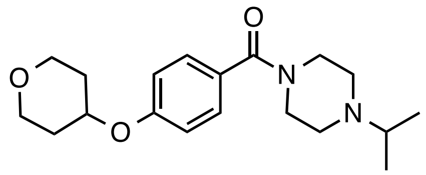

8 was prepared using General Procedure 1 in glassware ii. In chamber A was added Xantphos Pd G4 (24 mg, 0.025 mmol), 4-(4-iodophenoxy)tetrahydro-2*H*-pyran (152 mg, 0.5 mmol), dioxane (3 mL), Et_3N (139 μL, 1.0 mmol) and 1-isopropylpiperazine (203 μL, 1.0 mmol). The ElectroWare was set to produce 750 μmol CO. Chamber A was stirred at 80 °C. Column chromatography (pentane/EtOAc/Et_3N, 10:10:1) afforded a brown oil (131 mg, 79%). ^{1}H NMR (400 MHz, $CDCl_3$) δ 7.36 (d, J = 8.4 Hz, 2H), 6.90 (d, J = 8.5 Hz, 2H), 4.51 (tt, J = 7.6, 3.7 Hz, 1H), 4.00–3.92 (m, 2H), 3.83–3.37 (m, 6H), 2.71 (sept, J = 6.5 Hz, 1H), 2.50 (bs, 4H), 2.06–1.96 (m, 2H), 1.84–1.72 (m, 2H), 1.03 (d, J = 6.5 Hz, 6H). ^{13}C NMR (100 MHz, $CDCl_3$) δ 170.1, 158.3, 129.2 (2C), 128.3, 115.6 (2C), 71.6, 65.0 (2C), 54.6, 48.7 (bs, 2C), 42.7 (bs, 2C), 31.7 (2C), 18.4 (2C). HRMS $C_{19}H_{29}N_2O_3$ [M+H$^+$]: calculated 333.2173, found 333.2181.
The data were in accordance to literature.[5]

2-(3-(Trifluoromethyl)phenyl)-5-(4-(trifluoromethyl)phenyl)-1,3,4-oxadiazole (9)

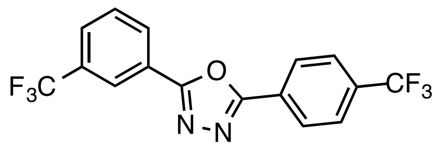

9 was prepared using General Procedure 1 and glassware ii. In chamber A was added 3-bromobenzotrifluoride (113 mg, 0.5 mmol), 4-(trifluoromethyl)benzohydrazide (102 mg, 0.53 mmol), Xantphos Pd G4 (24 mg, 0.025 mmol), dioxane (3 mL) and

Cy_2NMe (160 µL, 0.75 mmol). The ElectroWare was set to produce 750 µmol CO. Chamber A was stirred at 95 °C for 18 h. In a vial PPh_3 (262 mg, 1 mmol) and I_2 (254 mg, 1 mmol) was dissolved in dioxane and heated to 95 °C for 5 min before injected into the reaction mixture. Et_3N (279 µL) was injected afterwards and the reaction was stirred at 95 °C for additional 5 h. Column chromatography (pentane/CH_2Cl_2, 1:1) afforded a light yellow solid (131 mg, 73%). ^{1}H NMR (400 MHz, $CDCl_3$) δ 8.37 (s, 1H), 8.34 (d, J = 7.9 Hz, 1H), 8.26 (d, J = 8.2, 2H), 7.86 (m, 3H), 7.69 (t, J = 7.8 Hz, 1H). ^{13}C NMR (100 MHz, $CDCl_3$) δ 164.0 (2C), 133.7 (q, J = 33.0 Hz), 132.0 (q, J = 33.2 Hz), 130.3, 130.0 (2C), 128.7 (q, J = 3.6 Hz), 127.5 (2C), 126.9, 126.3 (q, J = 3.8 Hz), 124.5, 124.0 (q, J = 3.9 Hz), 123.6 (q, J = 272.6 Hz), 123.6 (q, J = 272.6 Hz). ^{19}F NMR (367 MHz, $CDCl_3$) δ –63.0, –63.2. HRMS $C_{16}H_9F_6N_2O$ [M+H$^+$]: calculated 359.0614, found 359.0619.
The data were in accordance to literature.[6]

N-(2-(Diethylamino)ethyl)-2-fluoronicotinamide (Melanoma PET tracer) (10)

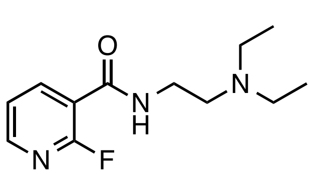

10 was prepared using General Procedure 1 in glassware i. In chamber A was added 2-fluoro-3-iodopyridine (112 mg, 0.5 mmol), Xantphos Pd G4 (24 mg, 0.025 mmol), dioxane (3 mL), Et_3N (139 µL, 1 mmol) and *N*,*N*-diethylethane-1,2-diamine (142 µL, 1 mmol). The ElectroWare was set to produce 750 µmol CO. Chamber A was stirred at rt. Column chromatography (pentane/EtOAc/Et_3N, 15:5:1) afforded a brown oil (99 mg, 83%). ^{1}H NMR (400 MHz, $CDCl_3$) δ 8.49 (ddd, J = 9.7, 7.5, 2.0 Hz, 1H), 8.30 (d, J = 4.6 Hz, 1H), 7.83 (bs, 1H), 7.33 (ddd, J = 7.2, 4.8, 2.1 Hz, 1H), 3.75 (q, J = 5.5 Hz, 2H), 3.04 (t, J = 6.1 Hz, 2H), 2.92 (q, J = 6.7 Hz, 4H), 1.25 (t, J = 7.2 Hz, 6H). ^{13}C NMR (100 MHz, $CDCl_3$) δ 162.7 (d, J = 7.0 Hz), 160.1 (d, J = 239.0 Hz), 150.3 (d, J = 16.0 Hz), 142.8 (d, J = 2.7 Hz), 122.2 (d, J = 4.4 Hz), 116.23 (d, J = 27.5 Hz), 51.3, 47.8 (2C), 36.4, 10.3 (2C). ^{19}F NMR (367 MHz, $CDCl_3$) δ –64.6. HRMS $C_{12}H_{19}FN_3O$ [M+H$^+$]: calculated 240.1507 found 240.1514.
The data were in accordance to literature.[7]

2-(Diethylamino)ethyl 4-butoxybenzoate (Butoxycaine) (11)

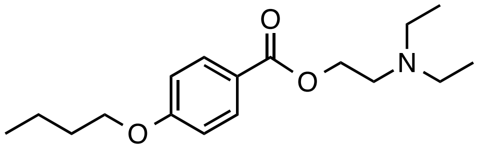

11 was prepared using General Procedure 1 in glassware ii. In chamber A was added Xantphos Pd G4 (24 mg, 0.025 mmol), 1-bromo-4-butoxybenzene (121 mg, 0.5 mmol), DMAP (15 mg, 0.125 mmol), Na_2CO_3 (160 mg, 1.5 mmol), toluene (3 mL) and 2-(diethylamino)ethan-1-ol (266 µL, 2 mmol). The ElectroWare was set to produce 750 µmol CO. Chamber A was stirred at 100 °C. Column chromatography (2% MeOH in DCM) afforded a colourless solid (144 mg, 98%). ^{1}H NMR (400 MHz, $CDCl_3$) δ 7.95 (d, J = 8.7 Hz, 2H), 6.88 (d, J = 8.8 Hz, 2H), 4.37 (t, J = 6.2 Hz, 2H), 3.98 (t, J = 6.5 Hz, 2H), 2.87 (t, J = 6.2 Hz, 2H), 2.66 (q, J = 7.1 Hz, 4H), 1.76 (quin, J = 6.6 Hz, 2H), 1.47, (sext, J = 7.4 Hz, 2H), 1.08 (t, J = 7.1 Hz, 6H), 0.96 (t, J = 7.4 Hz,3H). ^{13}C NMR (100 MHz, $CDCl_3$) δ 166.4, 163.1, 131.7 (2C), 122.4, 114.1 (2C), 68.0, 62.8, 51.1, 47.9 (2C), 31.2, 19.3, 13.9, 11.9 (2C). HRMS $C_{17}H_{28}NO_3$ [M+H$^+$]: calculated 294.2064, found 294.2075.
The data were in accordance to literature.[8]

Cyclopropyl methylquinoline-8-carboxylate (12)

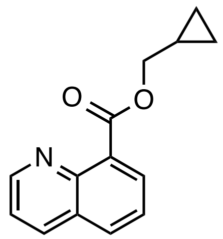

12 was prepared using General Procedure 2 and glassware ii. In chamber A was added quinolin-8-yl 4-methylbenzenesulfonate (150 mg, 0.5 mmol), $Pd(OAc)_2$ (4.5 mg, 0.02 mmol), dcpp•$2HBF_4$ (12 mg, 0.02), K_2CO_3 (138 mg, 1 mmol), crushed molecular sieves (200 mg), toluene (3 mL) and cyclopropylmethanol (108 mg, 1.5 mmol). The ElectroWare was set to produce 750 μmol CO. Chamber A was stirred at 90 °C. Column chromatography (20-30% EtOAc in pentane) afforded a colourless oil (78 mg, 69%). ^1H NMR (400 MHz, $CDCl_3$) δ 9.04 (dd, *J* = 4.1, 1.6 Hz, 1H), 8.16 (dd, *J* = 8.3, 1.6 Hz, 1H), 8.04 (dd, *J* = 7.1, 1.2 Hz, 1H), 7.91 (d, *J* = 7.3 Hz, 1H), 7.55 (t, *J* = 7.7 Hz, 1H), 7.43 (dd, *J* = 8.3, 4.2 Hz, 1H), 4.30 (d, *J* = 7.3 Hz, 2H), 1.31 (tt, *J* = 7.6, 4.8 Hz, 1H), 0.62 (q, *J* = 5.9 Hz, 2H), 0.41 (q, *J* = 4.8 Hz, 2H). ^{13}C NMR (100 MHz, $CDCl_3$) δ 167.9, 151.6, 145.7, 136.3, 132.0, 131.3, 130.4, 128.5, 125.7, 121.7, 70.4, 10.0, 3.5 (2C). HRMS $C_{14}H_{14}NO_2$ [M+H$^+$]: calculated 228.1019, found 228.1026.
The data were in accordance to literature.[9]

Isopropyl 2-(4-(4-chlorobenzoyl)phenoxy)-2-methylpropanoate (Fenofibrate) (13)

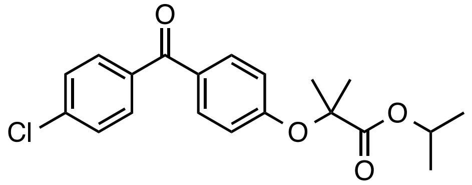

13 was prepared using General Procedure 2 and glassware ii. In chamber A was added isopropyl 2-(4-bromophenoxy)-2-methylpropanoate (151 mg, 0.5 mmol), 2-(4-chlorophenyl)-1,3,6,2-dioxazaborocane (124 mg, 0.55 mmol), $Pd(acac)_2$ (7.6 mg, 0.025 mmol), CataCXium A•HI (24 mg, 0.05 mmol), toluene (3 mL) and water (300 μL). The ElectroWare was set to produce 1.10 mmol CO. The CO was pre-released at rt before chamber A was heated and stirred at 80 °C for 18 h. Column chromatography (pentane/EtOAc, 25:1) afforded a brown oil (103 mg, 57%). ^1H NMR (400 MHz, $CDCl_3$) δ 7.72 (d, *J* = 8.8 Hz, 2H), 7.69 (d, *J* = 8.4 Hz, 2H), 7.43 (d, *J* = 8.4 Hz, 2H), 6.86 (d, *J* = 8.8 Hz, 2H), 5.08 (sept, *J* = 6.3 Hz, 1H), 1.65 (s, 6H), 1.19 (d, *J* = 6.3 Hz, 6H). ^{13}C NMR (100 MHz, $CDCl_3$) δ 194.3, 173.2, 159.8, 138.4, 136.5, 132.0 (2C), 131,2 (2C), 130.3, 128.6 (2C), 117.3 (2C), 79.5, 69.4, 25.5 (2C), 21.6 (2C). HRMS $C_{20}H_{22}ClO_4$ [M+H$^+$]: calculated 361.1201, found 361.1210.
The data were in accordance to literature.[10]

1-(Quinolin-3-yl)-3-(triisopropylsilyl)prop-2-yn-1-one (14)

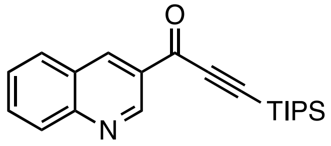

14 was prepared using General Procedure 1 and glassware ii. In chamber A was added Xantphos Pd G4 (24 mg, 0.025 mmol), 3-bromoquinoline (104 mg, 0.5 mmol), dioxane (3 mL), ethynyltriisopropylsilane (137 mg, 0.75 mmol) and Et_3N (209 μL, 1.5 mmol). The ElectroWare was set to produce 750 μmol CO. Chamber A was stirred at 80 °C. Column chromatography (5–10% EtOAc in pentane) afforded a yellow solid (154 mg, 91%). ^1H NMR (400 MHz, $CDCl_3$) δ 9.58 (s, 1H), 8.94 (s, 1H), 8.17 (d, *J* = 8.5 Hz, 1H), 7.94 (d, *J* = 8.1 Hz, 1H), 7.86 (t, *J* = 7.6 Hz, 1H), 7.64 (t, *J* = 7.5 Hz, 1H), 1.28-1.21 (m, 3H), 1.21-1.15 (m, 18H). ^{13}C NMR (100 MHz, $CDCl_3$) δ 175.9, 150.3, 149.7, 139.7, 132.6, 129.6, 129.3, 127.8,

126.8, 102.6, 100.2, 18.7 (6C), 11.2 (3C). HRMS $C_{21}H_{28}NOSi$ [M+H$^+$]: calculated 338.1935, found 338.1945.

*N*2, *N*6-bis(2-((2-aminoethyl)amino)ethyl)pyridine-2,6-dicarboxamide (15)

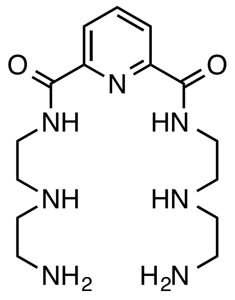

2,2-Diaminodiethylamine (52.6 g, 510 mmol) was added to a solution of dimethyl 2,6-pyridinedicarboxylate (10.0 g, 51.0 mmol) in dry methanol (800 mL) in a dried round bottom flask under argon. The reaction mixture was stirred for 24 h followed by refluxing for 1 h. Methanol was removed under reduced pressure and the excess 2,2-Diaminodiethylamine was removed under vacuum distillation at 50–52 °C at 180 mbar. This yielded **15** as a brown oil (14.2 g, 83%). ^{1}H NMR (400 MHz, $CDCl_3$) δ 8.51 (bs, 2H), 8.32 (d, *J* = 7.8 Hz, 2H), 7.99 (t, *J* = 7.8 Hz, 1H), 3.59 (q, *J* = 5.8 Hz, 4H), 2.89 (t, *J* = 5.8 Hz, 4H), 2.81 (t, *J* = 5.8 Hz, 4H), 2.71 (t, *J* = 5.8 Hz, 4H), 1.55 (bs, 6H). ^{13}C NMR (100 MHz, $CDCl_3$) δ 163.9, 149.1, 138.9, 125.0, 52.0, 48.8, 41.8, 39.6. HRMS $C_{15}H_{27}N_7O_2$ [M+H$^+$]: calculated 338.2299, found: 338.2304.
The data were in accordance to literature.[11]

Supplementary References

1) Amatore, C. A., Jutand, A. & F. Pflüger. Nanosecond time resolved cyclic voltammetry: Direct observation of electrogenerated intermediates with bimolecular diffusion controlled decay using scan rates in the megavolt per second range. *J. Electroanal. Chem.* **218**, 361–365 (1987).

2) Tinnis, F., Verho, O., Gustafson, K. P. J., Tai, C-W., Båckvall, J. E. & Adolfsson, H. Efficient Palladium-Catalyzed Aminocarbonylation of Aryl Iodides Using Palladium Nanoparticles Dispersed on Siliceous Mesocellular Foam. *Chem. Eur. J.* **20**, 5885–5889 (2014).

3) Zmuda, F., Malviya, G., Blair, A., Boyd, M., Chalmers, A. J., Sutherland, A. & Pimlott, S. L. Synthesis and Evaluation of a Radioiodinated Tracer with Specificity for Poly(ADP-ribose) Polymerase-1 (PARP-1) in Vivo *J. Med. Chem.* **58**, 8683–8693 (2015).

4) Elsinghorst, P. W. & Guetschow, M. Synthesis of ^{2}H- and ^{13}C-labelled sunitinib and its primary metabolite. *J. Label Compd. Radiopharm.* **52**, 360–365 (2009).

5) Hermange, P., Lindhardt, A. T., Taaning, R. H., Bjerglund, K., Lupp, D. & Skrydstrup, T. *Ex Situ* Generation of Stoichiometric and Substoichiometric ^{12}CO and ^{13}CO and Its Efficient Incorporation in Palladium Catalyzed Aminocarbonylations. *J. Am. Chem. Soc*. **133**, 6061–6071 (2011).

6) Andersen, T. L., Caneschi, W., Ayoub, A., Lindhardt, A. T., Couri, M. R. C. & Skrydstrup, T. 1,2,4- and 1,3,4-Oxadiazole Synthesis by Palladium-Catalyzed Carbonylative Assembly of Aryl Bromides with Amidoximes or Hydrazides. *Adv. Synth. Catal.* **356**, 3074–3082 (2014).

7) Nordeman, P., Friis, S. D., Andersen, T. L., Audrain, H., Larhed M., Skrydstrup, T. & Antoni, G. Rapid and Efficient Conversion of $^{11}CO_2$ to ^{11}CO through Silacarboxylic Acids: Applications in Pd-Mediated Carbonylations. *Chem. Eur. J*. **21**,17601–17604 (2015).

8) Gowrisankar, S., Sergeev, A. G, Anbarasan, P., Spannenberg, A., Neumann, H. & Beller, M. A General and Efficient Catalyst for Palladium-Catalyzed C-O Coupling Reactions of Aryl Halides with Primary Alcohols. *J. Am. Chem. Soc.* **132**, 11592–11598 (2010).

9) Friis, S. D., Taaning, R. H., Lindhardt, A. T. & Skrydstrup, T. Silacarboxylic Acids as Efficient Carbon Monoxide Releasing Molecules: Synthesis and Application in Palladium-Catalyzed Carbonylation Reactions. *J. Am. Chem. Soc.* **133**, 18114–18117 (2011).

10) Chu, L., Lipshultz, J. M. & Macmillan, D. W. C. Merging Photoredox and Nickel Catalysis: The Direct Synthesis of Ketones by the Decarboxylative Arylation of α-Oxo Acids. *Angew. Chem. Int. Ed.* **54**, 7929–7933 (2015).

11) Croucher, P. D., Klingele, M. H., Noble, A. & Brooker, S. Tricopper(II) complexes of unsymmetrical macrocycles incorporating phenol and pyridine moieties: The development of two stepwise routes. *Dalton Trans.* 4000–4007 (2007).
